# Cardiometabolic multimorbidity, social activity, and joint trajectories of physical disability, depressive symptom, and cognitive function in mid-to-late life: a multicohort study

**DOI:** 10.1186/s12916-025-04400-8

**Published:** 2025-10-22

**Authors:** Yue Zhang, Yaguan Zhou, Mika Kivimäki, Julianne Holt-Lunstad, Rodrigo M. Carrillo-Larco, Xiaolin Xu

**Affiliations:** 1 School of Public Health, The Second Affiliated Hospital, Zhejiang University School of Medicine, Hangzhou, Zhejiang 310058 China; 2 The Key Laboratory of Intelligent Preventive Medicine of Zhejiang Province, Hangzhou, China; 3 UCL Brain Sciences, University College London, London, UK; 4 Clinicum, University of Helsinki, Helsinki, Finland; 5 Department of Psychology, Brigham Young University, Provo, UT USA; 6 Emory Global Diabetes Research Center, Emory University, Atlanta, GA USA; 7 Hubert Department of Global Health, Rollins School of Public Health, Emory University, Atlanta, GA USA; 8 School of Public Health, Faculty of Medicine, The University of Queensland, Brisbane, Australia

**Keywords:** Cardiometabolic diseases, Cardiometabolic multimorbidity, Physical disability, Depressive symptom, Cognitive function, Social activity

## Abstract

**Background:**

There is limited evidence on the long-term associations between cardiometabolic multimorbidity (CMM) and multiple domains of functioning, and the potential role of social activity in these associations. We aimed to examine the associations between CMM and joint trajectories of physical disability, depressive symptom, and cognitive function, and whether social activity modifies these trajectories.

**Methods:**

This multicohort study used pooled data of four prospective cohorts of adults from China, the UK, the USA, and Europe. Participants with complete information on cardiometabolic diseases (CMDs, including heart diseases, stroke, and diabetes) and social activity at baseline were included. Physical disability, depressive symptom, and cognitive function were measured one to five times. Longitudinal modelling was used to describe post-baseline trajectories of the three domains, stratified by CMM status and social activity.

**Results:**

Among 73,778 participants (age 63.4 ± 9.1 years, 56.0% female), 20.0% had single CMD and 4.3% had CMM at baseline. Participants with single CMD or CMM had persistently worse physical disability, depressive symptom, and cognitive function compared to those without CMD. CMM was associated with faster worsening of physical disability (*β*_linear change*CMM_ = − 0.017 [95% confidence interval = − 0.030 to − 0.004]) and cognitive function (− 0.035 [− 0.050 to − 0.019]), particularly among those with social inactivity. Among 40,883 participants (age 62.9 ± 8.9 years, 55.6% female) in joint trajectory analysis, four trajectories were identified: ‘favourable trajectories of physical disability, depressive symptom, and cognitive function’ (48.1%); ‘worsening cognitive function’ (32.6%); ‘worsening depressive symptom and cognitive function’ (14.6%); and ‘rapidly-worsening physical disability and worsening depressive symptom and cognitive function’ (4.7%). CMD or CMM were associated with all the three worsening joint trajectories. The highest odds were observed for concurrent worsening across all three functional domains in participants with CMM (odds ratio 3.43 [2.80–4.20]), with more unfavourable trajectories in those with social inactivity (7.26 [5.49–9.59], *P* for additive interaction < 0.001).

**Conclusions:**

CMM was associated with worse levels, faster progression, and concurrent deterioration in physical disability, depressive symptom, and cognitive function, with the poorest trajectories among those who were socially inactive. This underscores the importance of implementing prevention strategies that integrate physical, psychological, cognitive, and social activities.

**Supplementary Information:**

The online version contains supplementary material available at 10.1186/s12916-025-04400-8.

## Background

The global burden of cardiometabolic diseases (CMDs), a group of interrelated disorders encompassing cardiovascular, metabolic, renal, and inflammatory systems, has been increasing during the past decades [1]. There were almost 20.5 million deaths related to cardiovascular diseases and 1.6 million deaths related to type 2 diabetes worldwide in 2021 [2, 3]. Concurrently, the prevalence of cardiometabolic multimorbidity (CMM), which is defined as the coexistence of two or more CMDs in an individual [4, 5], has also risen rapidly. There is evidence suggesting that each additional disease in CMM would double the risk of mortality, exaggerating the burden of single CMDs [5].

Observational studies have shown that CMM is associated with accelerated ageing as well as increased risks of falls, hospitalisation, and care home admission [6–8]. Individuals with CMM are more likely to experience physical limitation, depression, and cognitive impairment [9–12] and their longitudinal trajectories tend to worsen more rapidly over time [12–14]. For example, a prospective cohort study with 7-year follow-up suggested a faster progression to physical disability among individuals with CMM [13]. CMM has also been linked to a higher risk of depression among participants from East Asia, the USA, and Europe [12]. Another multicohort study combining data of 14 longitudinal cohort studies found a lower cross-sectional cognitive performance and a faster rate of cognitive decline in individuals with CMM [10]. In addition to separate changes of physical, psychological, and cognitive functioning following CMM, current evidence has suggested interrelationships between them [15]. For example, a reciprocal relationship between physical and cognitive functioning has been shown among older adults [16]. Bidirectional relationships between poor cognitive performance and worse psychological conditions were observed among populations from the UK, the USA, and China [17–19]. However, few studies have examined joint trajectories of physical disability, depressive symptom, and cognitive function. Given that CMM affects several organ systems, its impact on the concurrent deterioration of various functional capacities warrants further investigation.

Lifestyle factors, such as social activity, are known to influence trajectories of physical disability, depressive symptoms, and cognitive function [20–22]. Social activity refers to leisure pursuits undertaken for enjoyment or well-being, independent of work or daily living (e.g. playing cards, attending clubs) [23]. Participation in such activities may also be affected by health status, and existent evidence suggests that individuals with multimorbidity tend to engage less in social activities [24]. However, to our knowledge, no previous studies have examined whether social activity modifies the joint trajectories of physical disability, depressive symptoms, and cognitive function among individuals with CMDs or CMM.

In the current study, we combined data of four nationally representative cohort studies including populations from 18 countries. Our aim was to assess joint trajectories of physical disability, depressive symptom, and cognitive function among participants with or without CMM. In addition, we explored whether social activity modifies the association between CMM and trajectories of different functional domains.

## Methods

### Study design and population

The four prospective cohort studies included in this multicohort study were part of the Global Aging, Health, and Policy programme: the China Health and Retirement Longitudinal Study (CHARLS), the English Longitudinal Study on Ageing (ELSA), the US Health and Retirement Study (HRS), and the Survey of Health, Ageing and Retirement in Europe (SHARE). These studies are sister studies following similar study protocols, enabling the cross-regional comparisons [25]. Participants were interviewed every 2 or 3 years in each study where their information on sociodemographic characteristics, lifestyle behaviours, and health status were collected. Detailed study designs of the four cohorts are available elsewhere [26–29].

**Fig. 1:**
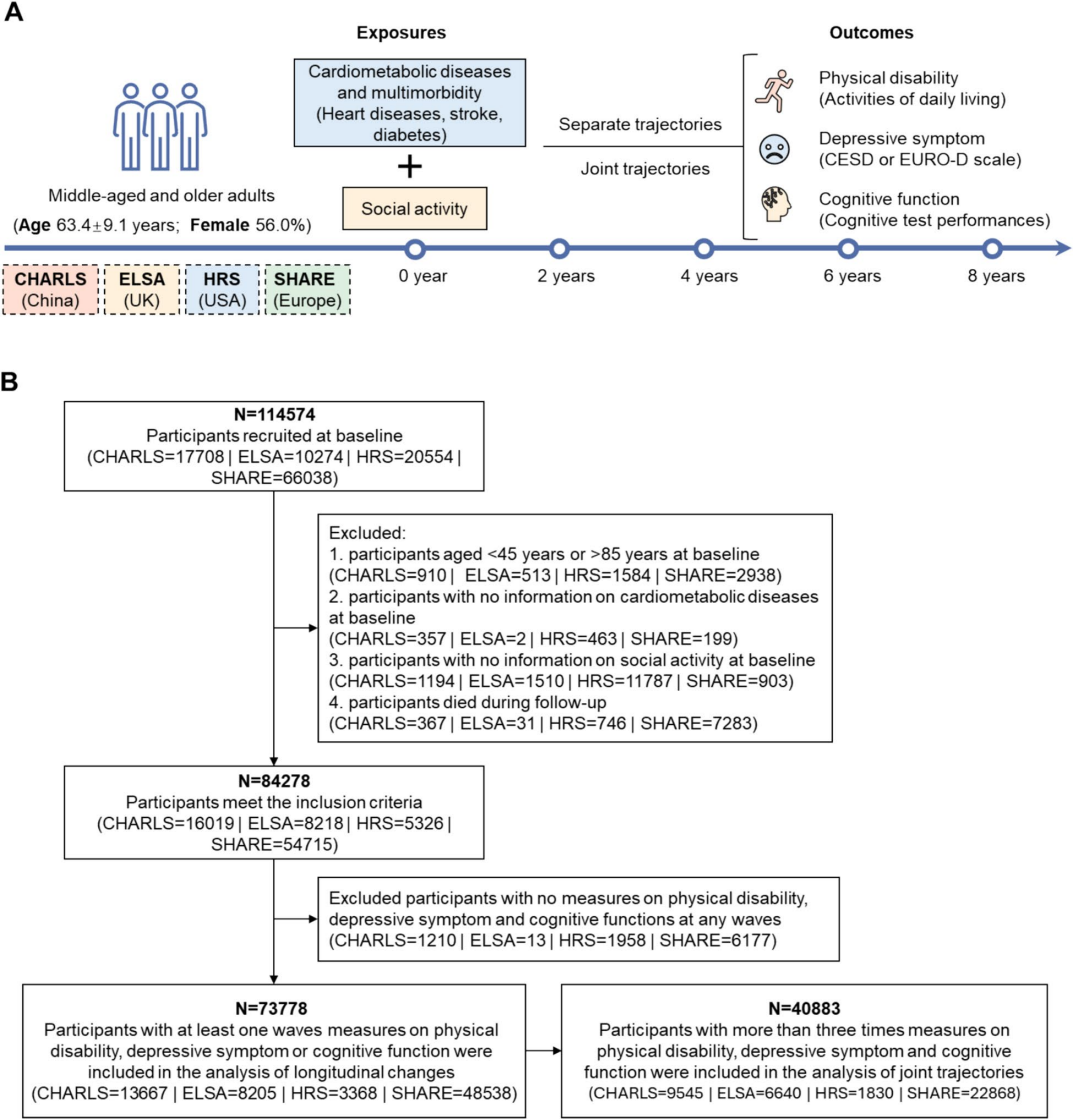
Study design and flow chart of the population selection process. **A** Diagram of the study design; **B** Population selection process. CHARLS, the China Health and Retirement Longitudinal Study; ELSA, the English Longitudinal Study on Ageing; HRS, the US Health and Retirement Study; SHARE, the Survey of Health, Ageing and Retirement in Europe

The study design and population selection process are presented in Fig. 1. To ensure consistent time periods across the four studies, we used data of five waves from each study (Additional file 1: Table S1): waves 1–5 of CHARLS (2011–2020), waves 5–9 of ELSA (2010–2019), waves 11–15 of HRS (2012–2021), and waves 5–9 of SHARE (2013–2022). We included participants aged 45–85 years who provided baseline information on CMDs and social activity, excluding those who died during follow-up. Participants with data from at least one wave of physical disability, depressive symptom, and cognitive function were included in the analysis of separate trajectories (*n* = 73,778). A subpopulation of participants with more than three repeated measures across these domains was further included in the analysis of joint trajectories of physical disability, depressive symptom, and cognitive function (*n* = 40,883). Baseline characteristics of participants included or not included in the analysis of joint trajectories are compared in Additional file 1: Table S2.

### Cardiometabolic diseases and multimorbidity at baseline

As in previous studies, the CMDs assessed in this study included diabetes, heart disease, and stroke [4, 11, 12]. The presence of these diseases was determined based on participants’ self-reported history. Heart disease was defined as participants reported the diagnosis of angina, heart attack, coronary heart disease, congestive heart failure, or other heart problems. The definition of stroke included stroke or cerebrovascular disease in ELSA and SHARE, while transient ischaemic attack was additionally included in HRS. Diabetes referred to diagnosed diabetes or high blood sugar. Detailed definitions of CMDs in the four studies are presented in Additional file 1: Table S3. We further defined CMM based on the number of individual CMDs, with the presence of two or more CMDs considered as CMM.

### Social activity at baseline

Social activity at baseline in CHARLS, HRS, and SHARE was determined based on participants’ self-reported engagements in activities in last month, such as voluntary work, educational or training courses, sport, social or other kind of club, and community-related organisation. Participants reporting an engagement at least once a month were considered to be socially active. In ELSA, participants reported whether they were members of any organisations, clubs, or societies; those reporting affirmatively were considered as socially active. Details of definitions in each study are shown in Additional file 1: Table S3.

### Physical disability, depressive symptom, and cognitive function at baseline and follow-up

The primary outcomes were longitudinal trajectories of physical disability, depressive symptom, and cognitive function over time. Physical disability was assessed based on participants’ reports on difficulties in performing activities of daily living (ADL), including dressing, bathing, eating, getting in or out bed, toileting, and walking across a room in ELSA, HRS, and SHARE. In CHARLS, the activity of walking across a room was not available; instead, an additional question about controlling urination and defecation was included. A summed score of ADLs was calculated to represent the level of physical disability, with a higher score denoting a worse physical disability.

Depressive symptom was measured through standardised questionnaire scales in each study. In HRS and ELSA, the 8-item version of the Center for Epidemiologic Studies Depression Scale (CESD-8) was used to measure depression. The 10-item version CESD scale was used to assess depression in CHARLS. In SHARE, a 12-item version of EURO-D scale was used to measure depression. A higher score of the depression scale represented a worse depressive symptom.

Cognitive function was measured through study-specific cognitive tests, including episodic memory (immediate and delayed word recall), orientation (a recall test of time, place, and person), executive (a serial 7’s subtraction test in CHARLS, HRS, and SHARE; an animal fluency test in SHARE), and visuospatial (picture drawing task only in CHARLS) performance. In CHARLS, the word list and the number of tests for immediate and delayed word recall tests were changed in 2018. We therefore used a weighted equipercentile equating method to adjust the word recall scores in 2018 and 2020 based on the percentile ranks observed in 2015 [30]. The composite cognitive scores represented the level of participants’ cognitive function, with a lower score representing a worse cognitive function.

To ensure consistency in the direction of scores across physical disability, depressive symptoms, and cognitive function, we reversed the ADL and depressive scores so that lower scores indicated worse functioning in all three domains. To compare the scores across four studies, we further standardised the baseline distribution of the three scores to have a mean of 0 and standard deviation (SD) of 1 (i.e. they were transformed into z-scores), and scores at follow-up surveys were standardised to the baseline distribution for comparability over time in each study [31]. Finally, standardised z-scores were derived for physical disability, depressive symptom, and cognitive function, where lower scores represented worse functional performances. Details of the measurement strategies for physical disability, depressive symptom, and cognitive function in each study are provided in Additional file 1: Table S3.

### Baseline covariates

Covariates assessed in this study included sociodemographic characteristics (age, sex, educational level, total household wealth, marital status), lifestyle factors (body mass index, current smoking status, alcohol consumption, and physical activity), and baseline comorbidities (hypertension, lung diseases, and cancer). Self-reported treatments or medications for hypertension, diabetes, and heart problems were also extracted for sensitivity analyses. Missing covariates were assigned to the ‘unknown’ groups. Further details of the harmonisation strategies for covariates are presented in Additional file 1: Table S3.

### Statistical analysis

Baseline characteristics of participants were described as mean (SD) for continuous variables and number (percentage) according to study and CMD status at baseline, and were compared using analysis of variance and chi-square tests as appropriate.

In the primary analysis, we used multilevel growth curve models to assess the associations of CMM and social activity with longitudinal changes of physical disability, depressive symptom, and cognitive function over time. Both linear and quadratic terms were included to allow for linear and nonlinear trajectories. Interaction terms of CMM and social activity with the growth parameters (i.e. linear and quadratic terms) were included in the models to account for potential differences in changing rates. Individuals and follow-up years were included as random effects in the models. Covariates included study, age at baseline, sex, educational level, total household wealth, marital status, body mass index, current smoking status, alcohol consumption, physical activity, baseline hypertension, lung diseases, and cancer.

In the secondary analysis, we measured the joint trajectories of physical disability, depressive symptom, and cognitive function over time. We used a group-based multi-trajectory modelling (GBMTM) approach to determine the concurrent trajectories of physical disability, depressive symptom, and cognitive function, which is a methodology allowing for the simultaneous analysis of multiple indicators. The optimal number of multi-trajectory groups were chosen based on Bayesian Information Criterion (BIC close to zero), the average posterior probabilities of assignment (Avepp > 0.7), and the sample sizes in each group [32] (Additional file 1: Table S4). We then used multinomial logistic regression models to assess the associations of CMM and social activity with identified joint trajectories of physical disability, depressive symptom, and cognitive function by estimating odds ratios (ORs) and 95% confidence intervals (CIs). To account for the heterogeneity across studies, meta-analyses based on random-effects models were conducted to summarise study-specific estimates for the associations of CMM and social activity with the joint trajectories, with the between-study heterogeneity tested using *I*^2^ statistics. We assessed both additive and multiplicative interactions of CMM and social activity on the joint trajectories using the delta method [33].

Several additional analyses were conducted to test the robustness of results. First, the longitudinal changes of physical disability, depressive symptom, and cognitive function by CMM and social activity were compared among participants within different cohorts. Second, subgroup analysis was performed to assess the associations of CMM and social activity with joint trajectories of physical disability, depressive symptom, and cognitive function stratified by sex and age. Third, sensitivity analyses for the association of CMM and social activity with separate and joint trajectories were conducted by excluding participants with missing covariates; imputing missing covariates using multiple imputation; including treatments or medications for hypertension, diabetes, and heart problems as covariates in the models; and redefining socially active as participants reported participating at least one social activities or groups in last month or year based on a previous study [34].

Significance tests were evaluated at the 0.05 level using two-sided tests. All statistical analyses were conducted using SAS (version 9.4) and R software (version 4.3.3). The PROC TRAJ procedure in SAS is used for the GBMTM method.

## Results

### Baseline characteristics of participants

The final analysis included 73,778 participants, of whom 13,667 (18.5%) were from China, 8205 (11.1%) from the UK, 3368 (4.6%) from the USA, and 48,538 (65.8%) from other European countries (Table 1). The mean (SD) age of participants at baseline was 63.4 (9.1) years. Among the included participants, 20.0% had a single CMD and 4.3% had CMM at baseline. The characteristics of participants by baseline CMM status are shown in Additional file 1: Table S5. Compared to participants with no CMD, those with a single CMD or CMM were more likely to be older, males, lower educated, with lower household wealth, widowed, overweight or obese, non-smokers, non-drinkers, physically inactive, socially inactive, and had higher rates of baseline hypertension, cancer, and lung diseases.

**Table 1:** Baseline characteristics of participants by different cohort studies

	**Total (*N* = 73,778)**	**CHARLS (*N* = 13,667)**	**ELSA (*N* = 8205)**	**HRS (*N* = 3368)**	**SHARE (*N* = 48,538)**
**Age at baseline, mean (SD)**	63.4 (9.1)	58.2 (8.9)	65.6 (8.5)	68.5 (7.7)	64.2 (8.7)
**Sex, *n* (%)**					
Male	32,437 (44.0)	6747 (49.4)	3665 (44.7)	1233 (36.6)	20,792 (42.8)
Female	41,341 (56.0)	6920 (50.6)	4540 (55.3)	2135 (63.4)	27,746 (57.2)
**Educational level, *n* (%)**					
Primary	20,005 (27.1)	8731 (63.9)	2190 (26.7)	558 (16.6)	8526 (17.6)
Secondary	37,390 (50.7)	4622 (33.8)	3877 (47.3)	1192 (35.4)	27,699 (57.1)
Tertiary	15,743 (21.3)	314 (2.3)	1498 (18.3)	1618 (48.0)	12,313 (25.4)
Unknown	640 (0.9)	0 (0.0)	640 (7.8)	0 (0.0)	0 (0.0)
**Total household wealth, *n* (%)**					
Q1 (lowest)	17,448 (23.6)	2454 (18.0)	2019 (24.6)	842 (25.0)	12,133 (25.0)
Q2	17,460 (23.7)	2460 (18.0)	2022 (24.6)	842 (25.0)	12,136 (25.0)
Q3	17,450 (23.7)	2455 (18.0)	2018 (24.6)	842 (25.0)	12,135 (25.0)
Q4 (highest)	17,451 (23.7)	2456 (18.0)	2019 (24.6)	842 (25.0)	12,134 (25.0)
Unknown	3969 (5.4)	3842 (28.1)	127 (1.5)	0 (0.0)	0 (0.0)
**Marital status, *n* (%)**					
Married or partnered	58,351 (79.1)	12,184 (89.1)	6048 (73.7)	2232 (66.3)	37,887 (78.1)
Widowed	7508 (10.2)	1216 (8.9)	994 (12.1)	575 (17.1)	4723 (9.7)
Separated or divorced or single	7866 (10.7)	267 (2.0)	1162 (14.2)	559 (16.6)	5878 (12.1)
Unknown	53 (0.1)	0 (0.0)	1 (0.0)	2 (0.1)	50 (0.1)
**Body mass index, *n* (%)**					
< 18.5, kg/m^2^	1259 (1.7)	696 (5.1)	46 (0.6)	29 (0.9)	488 (1.0)
18.5–24.9, kg/m^2^	27,275 (37.0)	7190 (52.6)	1808 (22.0)	811 (24.1)	17,466 (36.0)
25–29.9, kg/m^2^	27,092 (36.7)	3042 (22.3)	3029 (36.9)	1224 (36.3)	19,797 (40.8)
≥ 30, kg/m^2^	14,081 (19.1)	583 (4.3)	2260 (27.5)	1261 (37.4)	9977 (20.6)
Unknown	4071 (5.5)	2156 (15.8)	1062 (12.9)	43 (1.3)	810 (1.7)
**Current smoking status, *n* (%)**					
No	59,379 (80.5)	9353 (68.4)	7198 (87.7)	2979 (88.5)	39,849 (82.1)
Yes	14,371 (19.5)	4310 (31.5)	1007 (12.3)	372 (11.0)	8682 (17.9)
Unknown	28 (0.0)	4 (0.0)	0 (0.0)	17 (0.5)	7 (0.0)
**Alcohol consumption, *n* (%)**					
Less than weekly drinking	40,210 (54.5)	10,672 (78.1)	2714 (33.1)	2076 (61.6)	24,748 (51.0)
Weekly drinking or more	32,631 (44.2)	2286 (16.7)	5291 (64.5)	1286 (38.2)	23,768 (49.0)
Unknown	937 (1.3)	709 (5.2)	200 (2.4)	6 (0.2)	22 (0.0)
**Physical activity, *n* (%)**					
Physical inactive	14,822 (20.1)	9842 (72.0)	1142 (13.9)	530 (15.7)	3308 (6.8)
Physical active	58,953 (79.9)	3825 (28.0)	7063 (86.1)	2838 (84.3)	45,227 (93.2)
Unknown	3 (0.0)	0 (0.0)	0 (0.0)	0 (0.0)	3 (0.0)
**Baseline comorbidities, *n* (%)**					
Hypertension	29,590 (40.1)	3498 (25.6)	3418 (41.7)	2117 (62.9)	20,557 (42.4)
Cancer	5190 (7.0)	116 (0.8)	787 (9.6)	518 (15.4)	3769 (7.8)
Lung disease	5597 (7.6)	1295 (9.5)	451 (5.5)	308 (9.1)	3543 (7.3)
**Social activity, *n* (%)**					
Socially inactive	38,300 (51.9)	6892 (50.4)	2709 (33.0)	594 (17.6)	28,105 (57.9)
Socially active	35,478 (48.1)	6775 (49.6)	5496 (67.0)	2774 (82.4)	20,433 (42.1)
**Baseline cardiometabolic diseases, *n* (%)**					
No cardiometabolic disease	55,823 (75.7)	11,178 (81.8)	6041 (73.6)	1921 (57.0)	36,683 (75.6)
Single cardiometabolic disease	14,784 (20.0)	2154 (15.8)	1864 (22.7)	1107 (32.9)	9659 (19.9)
Cardiometabolic multimorbidity	3171 (4.3)	335 (2.5)	300 (3.7)	340 (10.1)	2196 (4.5)

### Longitudinal changes of physical disability, depressive symptom, and cognitive function

**Fig. 2:**
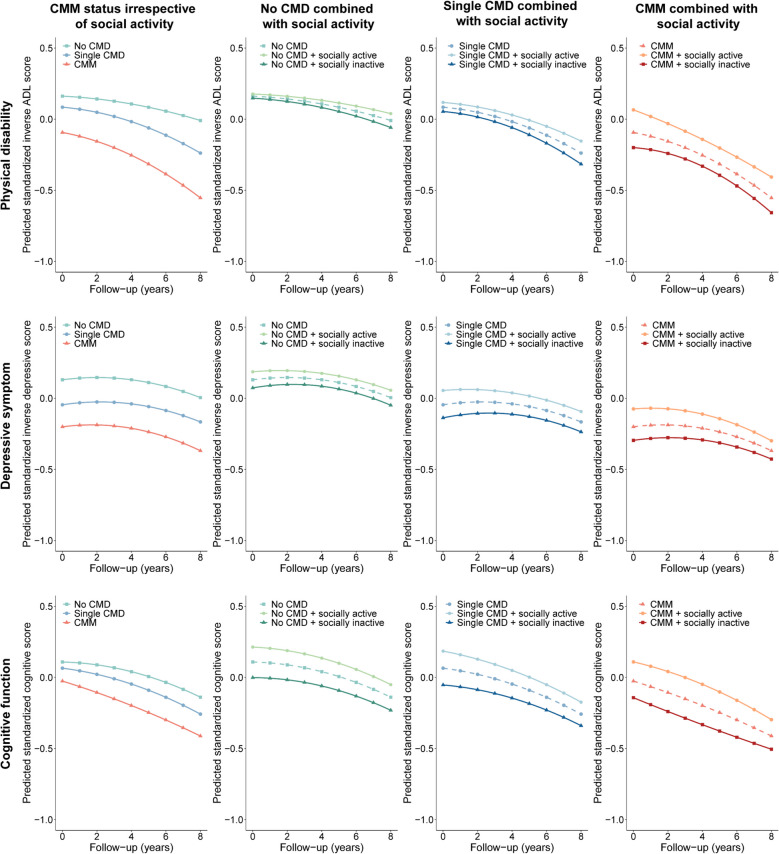
Estimated trajectories of physical disability, depressive symptom, and cognitive function during follow-up by cardiometabolic multimorbidity and social activity. Trajectories are adjusted for cohort, age at baseline, sex, educational level, total household wealth, marital status, body mass index, current smoking status, alcohol consumption, physical activity, baseline hypertension, lung diseases, and cancer. Lower values of inverse ADL score, inverse depressive score, and cognitive score indicated worsen physical disability, depressive symptoms, and cognitive function. ADL, activities of daily living; CMD, cardiometabolic disease; CMM, cardiometabolic multimorbidity

The observed separate trajectories of physical disability, depressive symptom, and cognitive function by CMM and social activity are shown in Fig. 2. Participants with a single CMD or CMM had worsen physical disability (*β*_single CMD*vs.*no CMD_ = − 0.078 [95% CI = − 0.091 to − 0.065], *β*_CMM*vs.*no CMD_ = − 0.255 [95% CI = − 0.280 to − 0.230]), severe depressive symptom (*β*_single CMD*vs.*no CMD_ = − 0.176 [95% CI = − 0.192 to − 0.159], *β*_CMM*vs.*no CMD_ = − 0.331 [95% CI = − 0.363 to − 0.298]), and lower cognitive function (*β*_single CMD *vs. *no CMD_ = − 0.043 [95% CI = − 0.060 to − 0.027], *β*_CMM *vs. *no CMD_ = − 0.134 [95% CI = − 0.166 to − 0.103]) at baseline, compared to those with no CMD (Table 2). Moreover, participants with CMM had a faster rate of worsening in physical disability initially (*β*_linear change * CMM_ = − 0.017, 95% CI = − 0.030 to − 0.004) and this worsening persisted at a higher rate over time (*β*_quadratic change * CMM_ = − 0.002, 95% CI = − 0.004 to − 0.001). Participants with CMM also had a steeper decrease in cognitive function initially (*β*_linear change * CMM_ = − 0.035, 95% CI = − 0.050 to − 0.019) but a flatter trajectory later (*β*_quadratic change * CMM_ = 0.002, 95% CI = 0 to 0.004), compared to those with no CMD. The depressive symptoms were continuously more severe among participants with CMM throughout the follow-up; however, we observed no significant difference in the rate of change over time between those with or without CMM. These longitudinal patterns did not differ between cohorts (Additional file 1: Figs. S1–S4).

**Table 2:** Association of cardiometabolic multimorbidity and social activity with separate trajectories of physical disability, depressive symptom, and cognitive function over time according to the multilevel growth curve model

	**Physical disability**		**Depressive symptom**		**Cognitive function**	
	***β* (95% CI)**	***P* value**	***β* (95% CI)**	***P* value**	***β* (95% CI)**	***P* value**
**CMM status at baseline**						
Time	** − 0.006 (− 0.009 to − 0.003)**	**< 0.001**	**0.016 (0.012 to 0.019)**	**< 0.001**	− 0.003 (− 0.006 to 0.000)	0.076
Time^2^	** − 0.002 (− 0.002 to − 0.002)**	**< 0.001**	** − 0.004 (− 0.004 to − 0.004)**	**< 0.001**	** − 0.003 (− 0.004 to − 0.003)**	**< 0.001**
Single CMD	** − 0.078 (− 0.091 to − 0.065)**	**< 0.001**	** − 0.176 (− 0.192 to − 0.159)**	**< 0.001**	** − 0.043 (− 0.060 to − 0.027)**	**< 0.001**
CMM	** − 0.255 (− 0.280 to − 0.230)**	**< 0.001**	** − 0.331 (− 0.363 to − 0.298)**	**< 0.001**	** − 0.134 (− 0.166 to − 0.103)**	**< 0.001**
Time × single CMD	− 0.004 (− 0.011 to 0.002)	0.181	0.002 (− 0.005 to 0.010)	0.541	** − 0.012 (− 0.020 to − 0.005)**	**0.001**
Time × CMM	** − 0.017 (− 0.030 to − 0.004)**	**0.011**	0 (− 0.016 to 0.016)	0.999	** − 0.035 (− 0.050 to − 0.019)**	**< 0.001**
Time^2^ × single CMD	** − 0.002 (− 0.003 to − 0.001)**	**< 0.001**	0 (− 0.001 to 0.001)	0.636	0 (− 0.001 to 0.001)	0.454
Time^2^ × CMM	** − 0.002 (− 0.004 to − 0.001)**	**0.002**	− 0.001 (− 0.003 to 0.001)	0.503	**0.002 (0.000 to 0.004)**	**0.048**
**CMM combined with social activity**						
Time	** − 0.005 (− 0.009 to − 0.001)**	**0.018**	**0.011 (0.006 to 0.016)**	**< 0.001**	** − 0.006 (− 0.010 to − 0.001)**	**0.014**
Time^2^	** − 0.029 (− 0.041 to − 0.018)**	**< 0.001**	** − 0.003 (− 0.004 to − 0.003)**	**< 0.001**	** − 0.003 (− 0.004 to − 0.003)**	**< 0.001**
No CMD + socially inactive	** − 0.029 (− 0.041 to − 0.018)**	**< 0.001**	** − 0.113 (− 0.128 to − 0.098)**	**< 0.001**	** − 0.215 (− 0.230 to − 0.200)**	**< 0.001**
Single CMD + socially active	** − 0.059 (− 0.078 to − 0.041)**	**< 0.001**	** − 0.131 (− 0.155 to − 0.108)**	**< 0.001**	** − 0.028 (− 0.052 to − 0.005)**	**0.016**
Single CMD + socially inactive	** − 0.123 (− 0.141 to − 0.106)**	**< 0.001**	** − 0.324 (− 0.346 to − 0.301)**	**< 0.001**	** − 0.267 (− 0.289 to − 0.244)**	**< 0.001**
CMM + socially active	** − 0.112 (− 0.151 to − 0.072)**	**< 0.001**	** − 0.261 (− 0.312 to − 0.211)**	**< 0.001**	** − 0.104 (− 0.153 to − 0.055)**	**< 0.001**
CMM + socially inactive	** − 0.377 (− 0.409 to − 0.345)**	**< 0.001**	** − 0.482 (− 0.524 to − 0.441)**	**< 0.001**	** − 0.356 (− 0.396 to − 0.316)**	**< 0.001**
Time × no CMD + socially inactive	− 0.002 (− 0.008 to 0.004)	0.487	**0.010 (0.003 to 0.017)**	**0.005**	0.005 (− 0.002 to 0.012)	0.135
Time × single CMD + socially active	− 0.005 (− 0.015 to 0.004)	0.280	− 0.001 (− 0.012 to 0.010)	0.886	** − 0.017 (− 0.028 to − 0.007)**	**0.001**
Time × single CMD + socially inactive	− 0.005 (− 0.014 to 0.003)	0.232	**0.014 (0.003 to 0.025)**	**0.012**	− 0.005 (− 0.015 to 0.006)	0.400
Time × CMM + socially active	** − 0.040 (− 0.06 to − 0.019)**	**< 0.001**	− 0.001 (− 0.025 to 0.023)	0.933	− 0.022 (− 0.045 to 0.000)	0.053
Time × CMM + socially inactive	− 0.003 (− 0.020 to 0.014)	0.708	0.007 (− 0.014 to 0.028)	0.508	** − 0.044 (− 0.066 to − 0.022)**	**< 0.001**
Time^2^ × no CMD + socially inactive	− 0.001 (− 0.001 to 0.000)	0.709	** − 0.001 (− 0.002 to 0.000)**	**0.008**	0 (− 0.001 to 0.001)	0.819
Time^2^ × single CMD + socially active	** − 0.001 (− 0.003 to 0.000)**	**0.011**	0 (− 0.002 to 0.001)	0.787	0.001 (− 0.001 to 0.002)	0.323
Time^2^ × single CMD + socially inactive	** − 0.003 (− 0.004 to − 0.002)**	**< 0.001**	− 0.001 (− 0.003 to 0.000)	0.062	0 (− 0.001 to 0.002)	0.754
Time^2^ × CMM + socially active	0 (− 0.003 to 0.002)	0.838	− 0.001 (− 0.004 to 0.002)	0.367	0.001 (− 0.002 to 0.004)	0.701
Time^2^ × CMM + socially inactive	** − 0.005 (− 0.007 to − 0.003)**	**< 0.001**	− 0.001 (− 0.004 to 0.002)	0.492	**0.004 (0.001 to 0.007)**	**0.014**

Covariates include cohort, age at baseline, sex, educational level, total household wealth, marital status, body mass index, current smoking status, alcohol consumption, physical activity, baseline hypertension, lung diseases, and cancer. The no CMD and no CMD combined with socially active were reference groups. Boldface indicates statistical significance.

*CMD* Cardiometabolic disease, *CMM* Cardiometabolic multimorbidity

Participants who were socially inactive also had consistently worse physical disability, depressive symptoms, and cognitive function initially and later on time, compared to those who were socially active (Additional file 1: Figs. S5 and S6). Moreover, irrespective of whether participants had CMDs or CMM at baseline, being socially inactive was associated with persistently worse levels of the three functioning (Fig. 2). In particular, when CMM was combined with social inactivity, the levels of physical disability (*β* = − 0.377, 95% CI = − 0.409 to − 0.345), depressive symptom (*β* = − 0.482, 95% CI = − 0.524 to − 0.441), and cognitive function (*β* = − 0.356, 95% CI = − 0.396 to − 0.316) at baseline were particularly worse (Table 2). Furthermore, participants with both CMM and social inactivity had a steeper worsening in physical disability over time (*β*_quadratic change*CMM+socially inactive_ = − 0.005, 95% CI = − 0.007 to − 0.003). The decline in cognitive functioning was also faster initially among participants with CMM and social inactivity (*β*_linear change*CMM+socially inactive_ = − 0.044, 95% CI = − 0.066 to − 0.022), although the decline in the trajectory was flatter later (*β*_quadratic change*CMM+socially inactive_ = 0.004, 95% CI = 0.001 to 0.007). These longitudinal changes did not differ between studies (Additional file 1: Figs. S1–S4). Similar results were found in sensitivity analyses (Additional file 1: Figs. S7–S9).

### Joint trajectories of physical disability, depressive symptom, and cognitive function

**Fig. 3:**
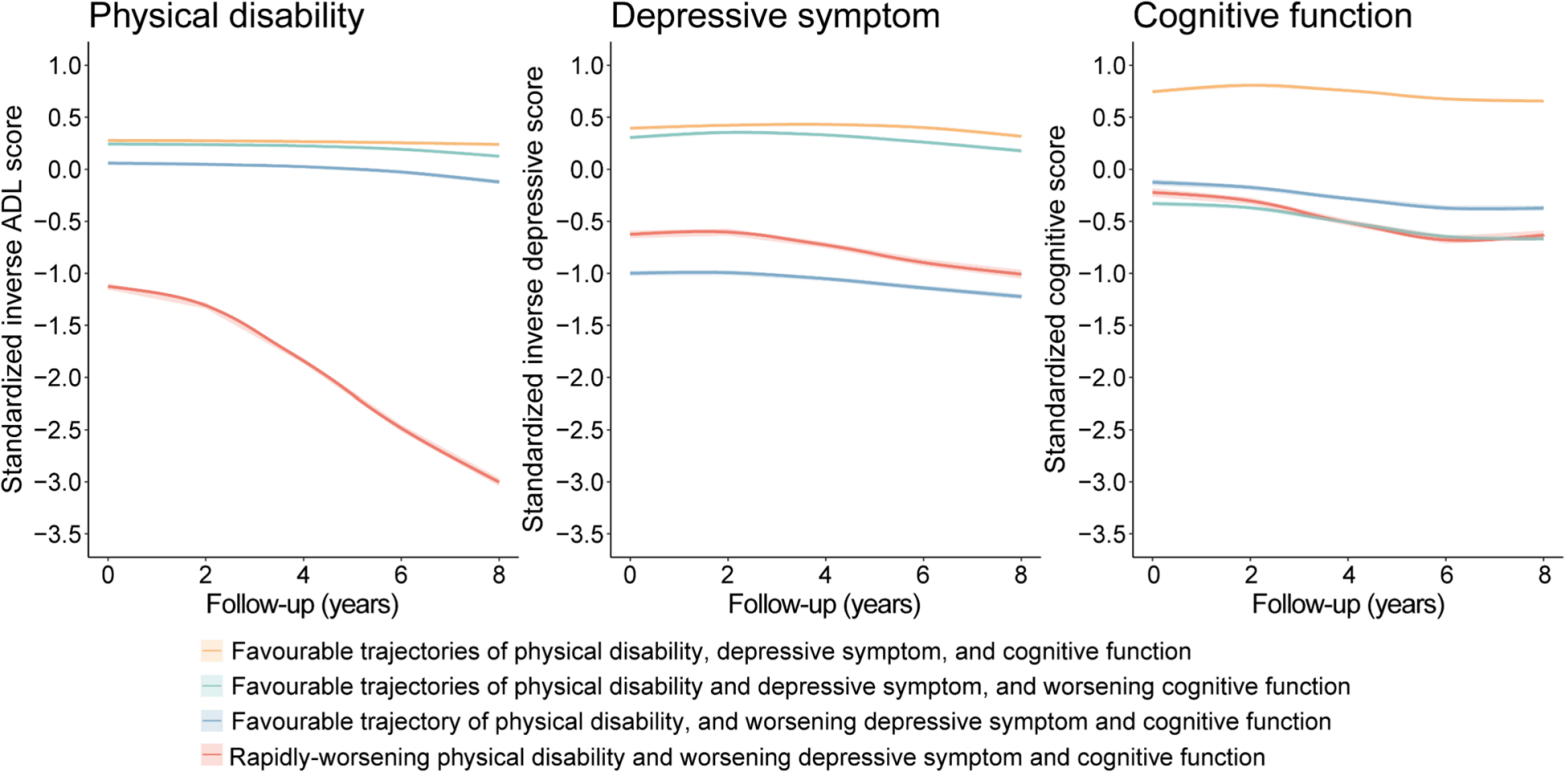
Four patterns of joint trajectories of physical disability, depressive symptom, and cognitive function. Lower values of inverse ADL score, inverse depressive score, and cognitive score indicated worse physical disability, depressive symptoms, and cognitive function

A total of 40,883 participants, with the mean (SD) age at baseline 62.9 (8.9) years, were included in the analysis of joint trajectories of physical disability, depressive symptom, and cognitive function (Additional file 1: Table S6). We identified four patterns of concurrently changing trajectories of physical disability, depressive symptom, and cognitive function (Fig. 3, Additional file 1: Table S7): ‘favourable trajectories of physical disability, depressive symptom, and cognitive function’ (*n* = 19,680, 48.1%); ‘favourable trajectories of physical disability and depressive symptom, and worsening cognitive function’ (*n* = 13,330, 32.6%); ‘favourable trajectory of physical disability, and worsening depressive symptom and cognitive function’ (*n* = 5949, 14.6%); and ‘rapidly-worsening physical disability and worsening depressive symptom and cognitive function’ (*n* = 1924, 4.7%).

Analysis linking CMM with joint trajectories showed that participants with a single CMD or CMM had higher odds of experiencing joint trajectories characterised by ‘favourable trajectories of physical disability and depressive symptom, and worsening cognitive function’, ‘favourable trajectory of physical disability and worsening depressive symptom and cognitive function’, and ‘rapidly-worsening physical disability and worsening depressive symptom and cognitive function’ (Table 3). The odds increased with the number of declining functioning domains, suggesting a higher likelihood of progressing toward overall functional deterioration for participants with CMDs. For example, among participants with CMM, the odds ranged from 1.34 (95% CI = 1.16–1.55) for experiencing only ‘worsening cognitive function’ to 3.43 (95% CI = 2.80–4.20) for experiencing a ‘rapidly-worsening physical disability and worsening depressive symptom and cognitive function’. There was no significant between-study heterogeneity in study-specific estimates (Additional file 1: Fig. S10).

**Table 3:** Association of cardiometabolic multimorbidity and social activity with joint trajectories of physical disability, depressive symptom, and cognitive function

	**Favourable trajectories of physical disability, depressive symptom, and cognitive function**	**Favourable trajectories of physical disability and depressive symptoms, and worsening cognitive function**	**Favourable trajectories of physical disability, and worsening depressive symptom and cognitive function**	**Rapidly-worsening physical disability and worsening depressive symptom and cognitive function**
**CMM status at baseline**				
No CMD	1.00 (reference)	1.00 (reference)	1.00 (reference)	1.00 (reference)
Single CMD	1.00 (reference)	**1.09 (1.02–1.17)**	**1.67 (1.54–1.80)**	**2.08 (1.85–2.34) **
CMM	1.00 (reference)	**1.34 (1.16–1.55)**	**2.55 (2.18–2.99)**	**3.43 (2.80–4.20)**
**CMM combined with social activity**				
No CMD + socially active	1.00 (reference)	1.00 (reference)	1.00 (reference)	1.00 (reference)
No CMD + socially inactive	1.00 (reference)	**1.50 (1.42–1.59)**	**1.74 (1.61–1.88)**	**1.99 (1.73–2.27)**
Single CMD + socially active	1.00 (reference)	**1.11 (1.02–1.21)**	**1.72 (1.54–1.93)**	**2.18 (1.83–2.58)**
Single CMD + socially inactive	1.00 (reference)	**1.60 (1.46–1.76)**	**2.80 (2.51–3.13)**	**3.96 (3.35–4.69)**
CMM + socially active	1.00 (reference)	**1.28 (1.05–1.56)**	**2.46 (1.96–3.08)**	**3.22 (2.39–4.34)**
CMM + socially inactive	1.00 (reference)	**2.13 (1.73–2.62)**	**4.66 (3.72–5.82)**	**7.26 (5.49–9.59)**

Covariates include cohort, age at baseline, sex, educational level, total household wealth, marital status, body mass index, current smoking status, alcohol consumption, physical activity, baseline hypertension, lung diseases, and cancer. For the model of baseline CMM, social activity was additionally adjusted

Odds ratios and 95% confidence intervals were reported in the table. Boldface indicates statistical significance.

*CMD* Cardiometabolic disease, *CMM* Cardiometabolic multimorbidity

**Fig. 4:**
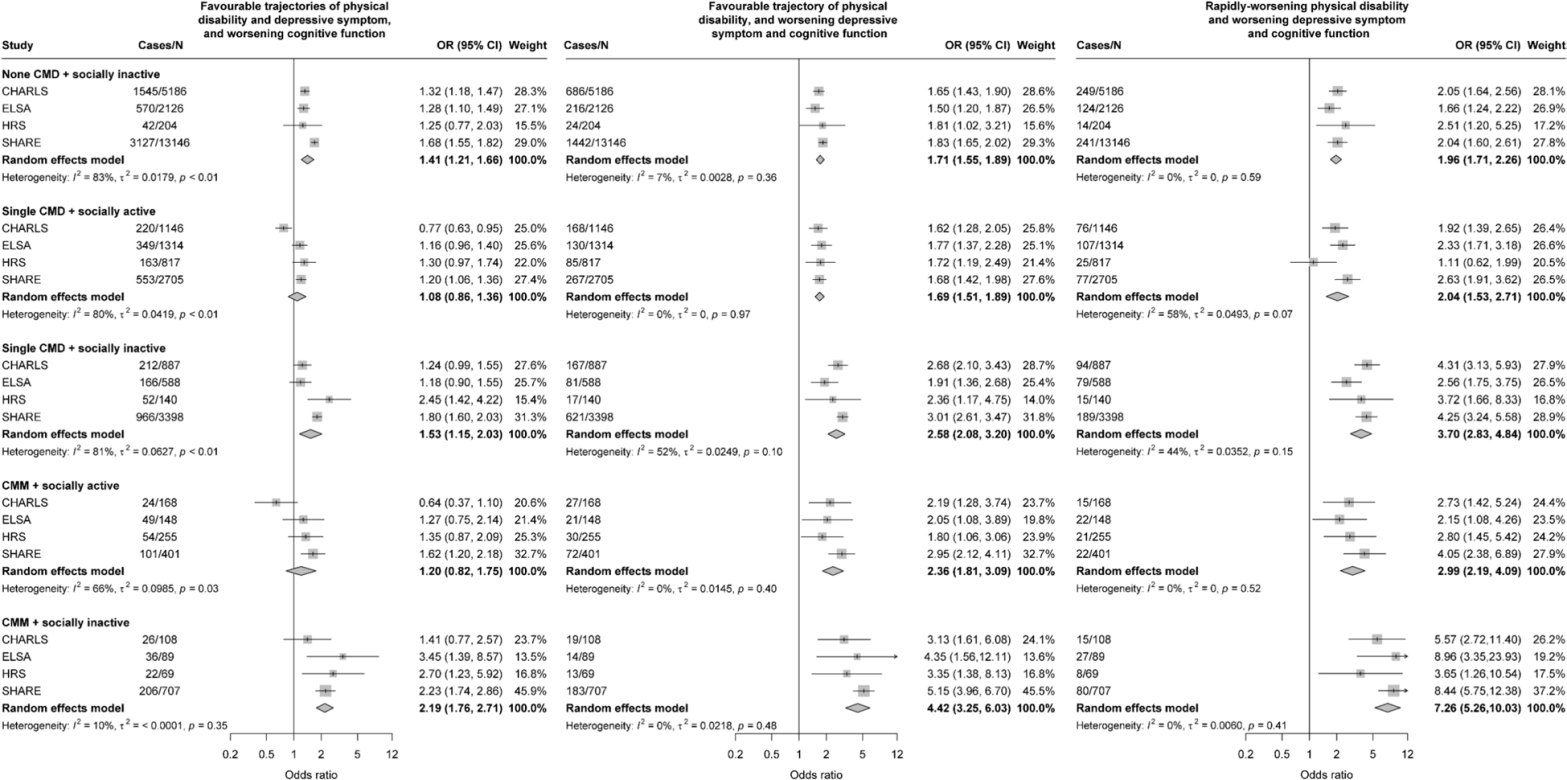
Random effect meta-analyses for the associations of CMM and social activity with joint trajectories of physical disability, depressive symptom, and cognitive function. The trajectory of favourable trajectories of physical disability, depressive symptom, and cognitive function, and no CMD combined with socially active are the reference groups. Covariates include age at baseline, sex, educational level, total household wealth, marital status, body mass index, current smoking status, alcohol consumption, physical activity, baseline hypertension, lung diseases, and cancer

These associations were more pronounced when CMM was combined with social inactivity (Table 3). Among participants with both CMM and social inactivity, the odds ranged from 2.13 (1.73–2.62) for experiencing ‘worsening cognitive function’ trajectory to 7.26 (95% CI = 5.49–9.59) for experiencing ‘rapidly-worsening physical disability and worsening depressive symptom and cognitive function’. In meta-analyses (Fig. 4), the pooled odds of the three trajectories characterised by only a worsening cognitive function, concurrent worsening depressive symptom and cognitive function, and rapidly-worsening physical disability and worsening depressive symptom and cognitive function were 2.19 (95% CI = 1.76–2.71), 4.42 (95% CI = 3.25–6.03), and 7.26 (95% CI = 5.26–10.03), respectively. We observed additive interaction effects between CMM and social activity on the three worsening trajectories, while multiplicative interaction effects were not observed (Additional file 1: Table S8). These associations were consistently observed across subpopulations stratified by sex and age (Additional file 1: Table S9, Additional file 1: Table S10). Similar results were found in sensitivity analyses (Additional file 1: Tables S11–S14).

## Discussion

### Principal findings

In this multicohort study of participants from 18 countries across Asia, North America, and Europe, we found that individuals with either a single CMD or CMM had persistently worse physical disability, depressive symptom, and cognitive function, both cross-sectionally and longitudinally. In particular, physical disability and cognitive function worsened faster among those with a CMD or CMM, although no significant difference in the rate of change in depressive symptom was observed. When assessing joint trajectories across the three domains of functioning, participants with CMM had 1.3 times higher odds of experiencing worsening cognitive function compared to those with no CMD. The corresponding odds ratio being 3.4 for the trajectory characterised by rapidly-worsening physical disability and worsening depressive symptom and cognitive function. We observed additive interaction effects between CMM and social activity on the joint trajectories. When CMM was combined with social inactivity, participants exhibited even poorer trajectories of physical disability, depressive symptom, and cognitive function, along with significantly steeper rates of worsening.

### Comparison with previous research

The observed cross-sectional and longitudinal associations of CMM with physical disability, depressive symptom, and cognitive function in our study were consistent with previous studies. For physical disability, CMM has been associated with greater levels of dysfunction and more rapid decline in populations from China, Canada, Ghana, and South Africa [13, 35, 36]. Unlike previous studies that applied linear models to assess longitudinal changes, we accounted for both linear and nonlinear trajectories. Our findings show that participants with CMM experienced steeper declines both at the early stages and later over time, compared to those without CMD. These results suggest that clinical monitoring and interventions for individuals with CMDs should begin early and be sustained over time, with particular attention to periods of accelerated functional decline.

Regarding psychological functioning, previous research has shown that individuals with CMM are at increased risk of depression and experience more severe depressive symptoms [12, 37–39]. However, little is known about the long-term trajectories and changes in depressive symptoms over time in this population. In our earlier study using group-based trajectory modelling, we identified three trajectories of depressive symptoms across a multi-country population from Asia, North America, and Europe: ‘persistently symptom-free’, ‘persistently low’, and ‘increasing’ [12]. CMM was associated with greater risk of both ‘persistently low’ and ‘increasing’ symptom trajectories. In the current study, we expanded these findings by examining depressive symptom trajectories separately for individuals with no CMD, a single CMD, and CMM. Both single CMD and CMM were associated with persistently severe depressive symptom. Although no significant differences in the rate of change over time was observed, these findings emphasise the importance of continuous mental health monitoring in the management of CMM.

Our findings also support previous studies reporting associations between CMM, lower cognitive performance and a faster rate of cognitive decline, compared to those with no CMD [10, 11, 40]. Additionally, existing evidence links CMM with an increased risk of developing dementia [41]. In our study, both individuals with a single CMD and those with CMM showed persistently lower levels of cognitive functioning. By modelling both linear and nonlinear changes, we observed that participants with CMM experienced a rapid decline in cognitive functioning during the early phase, followed by a slower decline over time. These findings highlight the importance of initiating cognitive screening and preventive strategies as soon as CMDs are diagnosed.

Using analysis of simultaneous changes of physical disability, depressive symptom, and cognitive function, we extended previous research by accounting for their interrelationships. We identified four distinct multi-domain trajectories, with the highest odds observed among CMM for the trajectory characterised by concurrent declines across all three functional domains. To date, few studies have applied joint trajectory modelling to examine changes across multiple functional domains in relation to specific diseases. A prospective cohort study modelled joint trajectories of physical frailty and cognition and found that rapid progression across both domains had an increased risk of all-cause mortality [42]. However, to our knowledge, no prior study has modelled the joint trajectories of physical disability, depressive symptom, and cognitive function simultaneously following the onset of CMDs or CMM. Our findings highlight the need for an integrated care strategy that simultaneously address physical, psychological, and cognitive domains of functioning concurrently in individuals with CMM.

The simultaneous worsening of physical disability, depressive symptom, and cognitive function observed among individuals with CMM are biologically plausible. CMM is often associated with chronic systemic inflammation [43], which can accelerate neurodegeneration [44], contribute to sarcopenia and frailty [45], and increase the risk of depressive symptoms [46, 47]. Furthermore, microvascular dysfunctions common in CMM may result in reduced cerebral perfusion and impaired muscle oxygenation, both of which are related to cognitive decline, sarcopenia, and mood disorders [48]. Genetic studies have also identified significant genetic correlations between cognitive condition and a range of physical and mental traits [49], supporting the interrelationships between physical, psychological, and cognitive functions in humans.

We observed social inactivity as a potential effect modifier of the association between CMM and trajectories of physical disability, depressive symptom, and cognitive function. Importantly, the rate of worsening in physical disability in individuals with both CMM and social inactivity was almost twice as fast as that observed in those with CMM alone. Accordingly, individuals with CMM had 3.4 times higher odds of experiencing concurrent decline across all three functional domains compared to those without CMD; this increased to 7.3-fold when CMM was combined with social inactivity.

The protective role of social activity in healthy ageing has been previously recognised. Social activities may promote mobility and encourage leisure-time physical activity, both of which support the maintenance of physical function [50]. Participating in social activities has also been associated with reduced negative emotions, improved self-esteem, and greater psychological well-being [51]. Additionally, social interactions can stimulate cognitive processes through communication, memory recall, attention, and problem-solving [52]. Our study expands these findings by demonstrating that social activity is not only independently associated with functional capacities, but may also buffer the adverse effects of CMM. This suggests a synergistic interaction between social and medical vulnerabilities in accelerating functional declines. These findings highlight the potential value of promoting social engagement as part of intervention strategies to slow multi-functional deterioration in the management of CMM. However, individuals with CMM may not have the ability to be socially active due to health conditions, especially traditional in-person activities. Digital interventions could be useful in these people and be considered in intervention strategies.

### Strengths and limitations

The strengths of this study include a large sample of participants from 18 middle- and high-income countries, the longitudinal measurements of multiple functional domains, the inclusion of both linear and nonlinear changes of functional levels over time, the consideration of interplays between different functional domains, and the application of multi-trajectory models for multiple indicators. Several limitations should be acknowledged. First, CMDs were self-reported at baseline, which may introduce recall bias. CMDs may also progress over time, which cannot be observed in this study. Additionally, multimorbidity assessment may affected by detection bias, whereby diagnosis of one disease increases the likelihood of detecting others due to heightened medical attention [11]. Second, we did not include hypertension in the definition of CMM, given that dichotomising elevated blood pressure may underestimate the true effect of blood pressure on chronic disease, as blood pressure has a continuous log-linear relationship with the risk of cardiovascular diseases throughout its range of values, and it is usually considered as a risk factor for CMDs and CMM [5, 53]. Third, definitions of physical disability, depressive symptom, and cognitive function varied across studies due to differences in measurement tools. To address this, we conducted cohort-specific analyses, which yielded conclusions consistent with those from pooled data. We also standardised the three measures across the five surveys within each study to enable cross-database comparisons. Fourth, the measurement of social activity varied across the four cohorts and ‘socially active’ was defined by monthly engagement in activities. Since no consensus exists on the threshold of social activity, the binary category is crude and may overlook the frequency and duration of participation. To address this issue, we conducted a sensitivity analysis redefining ‘socially active’ as participating at least one social activities or groups in last month or year following a previous study [20]. While associations between social activity and functional declines were consistent, future studies using standardised measurement protocols is needed. Fifth, as the study population was largely from upper-middle and high-income countries, generalisability to lower-income settings is limited. Sixth, participants died during follow-up were excluded from the analysis because we cannot observe their continuous trajectories. However, these individuals have significantly higher rates of CMDs and comorbidities (hypertension, lung disease, cancer) at baseline (Additional file 1: Table S15). Serious illness related to mortality makes multimorbidity and functional decline more likely, thus excluding these individuals may underestimate the true relationships. Besides, baseline characteristics also differed between participants included and excluded from the joint trajectory analyses, suggesting potential selection bias and limiting the representativeness and generalisability of our results. Lastly, despite adjusting for multiple covariates, the possibility of residual confounding cannot be excluded in observational studies. Also, the observational study design cannot provide evidence of causal relationships; intervention studies are needed before firm policy recommendations.

### Implications

Our study has important implications. In clinical practice, healthcare providers (e.g. geriatricians and general practitioners) are suggested to consider physical capacity, mental health, and cognitive performance simultaneously when assessing and managing patients with chronic diseases, especially for older adults with CMM. In particular, early prevention and intervention measures targeting these functions should be implemented upon the diagnosis of the first CMD to avoid further accelerated functional declines. When tailoring intervention programmes, social prescribing initiatives [22], which provide individuals with nonmedical sources of support (e.g. voluntary work, educational or training courses, sport, social or other kind of club, community-related organisation), can be incorporated as an additional approach [54]. For public health policies, our findings support the need to shift from a single-disease, physical capacity-oriented approach toward an integrated framework including psychological and cognitive functions, and the role of social engagement when managing CMM [55].

## Conclusions

This study showed that CMM is associated with worse levels, faster progression, and concurrent deterioration in physical disability, depressive symptom, and cognitive function, with the poorest trajectories observed for individuals with CMM who were also socially inactive. These findings support the need to move beyond single-disease, organ-specific model toward an integrated framework that incorporates multiple domains of functioning in the management of CMM.

## Supplementary Information

Additional file 1: Table S1 Countries, waves, time periods, and sample sizes included in present analyses according to studies. Table S2 Baseline characteristics of participants stratified by whether included in the analysis of joint trajectories. Table S3 Harmonised strategies for key variables included in the study. Table S4 Joint trajectory models’ results of model fitting process. Table S5 Baseline characteristics of participants included in the analysis of longitudinal changes of physical disability, depressive symptom, and cognitive function by baseline cardiometabolic diseases. Table S6 Baseline characteristics of participants included in the analysis of joint trajectories by baseline cardiometabolic diseases. Table S7 Baseline characteristics of participants by identified joint trajectories of physical disability, depressive symptom, and cognitive function. Table S8 Addictive and multiplicative estimates of the modification effect of social activity for the association between CMM and joint trajectories. Table S9 Association of cardiometabolic multimorbidity and social activity with joint trajectories of physical disability, depressive symptom, and cognitive function stratified by sex. Table S10 Association of cardiometabolic multimorbidity and social activity with joint trajectories of physical disability, depressive symptom, and cognitive function stratified by age. Tables S11–S14 Sensitivity analysis for the association of cardiometabolic multimorbidity and social activity with joint trajectories of physical disability, depressive symptom, and cognitive function. Table S15 Baseline characteristics of participants according to whether died during follow-up. Figs. S1–S4 Estimated trajectories of physical disability, depressive symptom, and cognitive function during follow-up by cardiometabolic multimorbidity and social activity in CHARLS, ELSA, HRS, and SHARE. Fig. S5 Predicted values of physical disability, depressive symptom, and cognitive function during follow-up stratified by social activity among the combined population. Fig. S6 Estimated trajectories of physical disability, depressive symptom, and cognitive function during follow-up by cardiometabolic multimorbidity and social activity across different cohorts. Figs. S7–S9 Sensitivity analysis for estimated trajectories of physical disability, depressive symptoms, and cognitive function during follow-up by cardiometabolic multimorbidity and social activity. Fig. S10 Random effect meta-analyses for the association of CMM with the joint trajectories of physical disability, depressive symptom, and cognitive function.

## Data Availability

The original data for this study are available on their respective websites: The China Health and Retirement Longitudinal Study (CHARLS; https://charls.pku.edu.cn/en/), the English Longitudinal Study on Ageing (ELSA; https://www.elsa-project.ac.uk), the Health and Retirement Study (HRS; https://hrs.isr.umich.edu), and the Survey of Health, Ageing and Retirement in Europe (SHARE; https://share-eric.eu).

## Abbreviations

ADL: Activities of daily living
CESD: The Center for Epidemiologic Studies Depression Scale
CHARLS: The China Health and Retirement Longitudinal Study
CI: Confidence interval
CMD: Cardiometabolic disease
CMM: Cardiometabolic multimorbidity
ELSA: The English Longitudinal Study on Ageing
HRS: The US Health and Retirement Study
OR: Odds ratio
SD: Standard deviation
SHARE: The Survey of Health, Ageing and Retirement in Europe
