# Supplementary material for: Cardiometabolic multimorbidity, social activity, and joint trajectories of physical disability, depressive symptom, and cognitive function in mid-to-late life: a multicohort study
Source: BMC Med. 2025 Oct 22;23:577. doi: 10.1186/s12916-025-04400-8 (PMC12542226; doi:10.1186/s12916-025-04400-8)
Supplement: Supplementary file 1 — Additional file 1: Table S1 Countries, waves, time periods, and sample sizes included in present analyses according to studies. Table S2 Baseline characteristics of participants stratified by whether included in the analysis of joint trajectories. Table S3 Harmonised strategies for key variables included in the study. Table S4 Joint trajectory models’ results of model fitting process. Table S5 Baseline characteristics of participants included in the analysis of longitudinal changes of physical disability, depressive symptom, and cognitive function by baseline cardiometabolic diseases. Table S6 Baseline characteristics of participants included in the analysis of joint trajectories by baseline cardiometabolic diseases. Table S7 Baseline characteristics of participants by identified joint trajectories of physical disability, depressive symptom, and cognitive function. Table S8 Addictive and multiplicative estimates of the modification effect of social activity for the association between CMM and joint trajectories. Table S9 Association of cardiometabolic multimorbidity and social activity with joint trajectories of physical disability, depressive symptom, and cognitive function stratified by sex. Table S10 Association of cardiometabolic multimorbidity and social activity with joint trajectories of physical disability, depressive symptom, and cognitive function stratified by age. Tables S11–S14 Sensitivity analysis for the association of cardiometabolic multimorbidity and social activity with joint trajectories of physical disability, depressive symptom, and cognitive function. Table S15 Baseline characteristics of participants according to whether died during follow-up. Figs. S1–S4 Estimated trajectories of physical disability, depressive symptom, and cognitive function during follow-up by cardiometabolic multimorbidity and social activity in CHARLS, ELSA, HRS, and SHARE. Fig. S5 Predicted values of physical disability, depressive symptom, and cognitive function during [file 12916_2025_4400_MOESM1_ESM.docx]

Supplementary information

[Supplementary Tables 3](#_Toc207380634)

[**Table S1.** Countries, waves, time periods and sample sizes included in present analyses according to studies. 3](#_Toc207380635)

[**Table S2.** Baseline characteristics of participants stratified by whether included in the analysis of joint trajectories. 4](#_Toc207380636)

[**Table S3.** Harmonized strategies for key variables included in the study. 6](#_Toc207380637)

[**Table S4.** Joint trajectory models’ results of model fitting process. 10](#_Toc207380638)

[**Table S5.** Baseline characteristics of participants included in the analysis of longitudinal changes of physical disability, depressive symptom, and cognitive function by baseline cardiometabolic diseases. 11](#_Toc207380639)

[**Table S6.** Baseline characteristics of participants included in the analysis of joint trajectories of physical disability, depressive symptom, and cognitive function by baseline cardiometabolic diseases. 13](#_Toc207380640)

[**Table S7.** Baseline characteristics of participants by identified joint trajectories of physical disability, depressive symptom, and cognitive function. 15](#_Toc207380641)

[**Table S8.** Addictive and multiplicative estimates of the modification effect of social activity for the association between CMM and joint trajectories of physical disability, depressive symptom, and cognitive function. 18](#_Toc207380642)

[**Table S9.** Association of cardiometabolic multimorbidity and social activity with joint trajectories of physical disability, depressive symptom, and cognitive function stratified by sex. 19](#_Toc207380643)

[**Table S10.** Association of cardiometabolic multimorbidity and social activity with joint trajectories of physical disability, depressive symptom, and cognitive function stratified by age. 20](#_Toc207380644)

[**Table S11.** Sensitivity analysis for the association of cardiometabolic multimorbidity and social activity with joint trajectories of physical disability, depressive symptom, and cognitive function by excluding participants with missing covariates. 22](#_Toc207380645)

[**Table S12.** Sensitivity analysis for the association of cardiometabolic multimorbidity and social activity with joint trajectories of physical disability, depressive symptom, cognitive function by imputing missing covariates. 23](#_Toc207380646)

[**Table S13.** Sensitivity analysis for the association of cardiometabolic multimorbidity and social activity with joint trajectories of physical disability, depressive symptom, and cognitive function by including treatments or medications for hypertension, diabetes and heart problems in the models. 24](#_Toc207380647)

[**Table S14.** Sensitivity analysis for the association of cardiometabolic multimorbidity and social activity with joint trajectories of physical disability, depressive symptom, and cognitive function by redefining social activity as respondents participated at least one social activities or groups in last month or year. 25](#_Toc207380648)

[**Table S15.** Baseline characteristics of participants according to whether died during follow-up. 26](#_Toc207380649)

[Supplementary Figures 28](#_Toc207380650)

[**Figure S1.** Estimated trajectories of physical disability, depressive symptom, and cognitive function during follow-up by cardiometabolic multimorbidity and social activity in CHARLS. 28](#_Toc207380651)

[**Figure S2.** Estimated trajectories of physical disability, depressive symptom, and cognitive function during follow-up by cardiometabolic multimorbidity and social activity in ELSA. 29](#_Toc207380652)

[**Figure S3.** Estimated trajectories of physical disability, depressive symptom, and cognitive function during follow-up by cardiometabolic multimorbidity and social activity in HRS. 30](#_Toc207380653)

[**Figure S4.** Estimated trajectories of physical disability, depressive symptom, and cognitive function during follow-up by cardiometabolic multimorbidity and social activity in SHARE. 31](#_Toc207380654)

[**Figure S5.** Predicted values of physical disability, depressive symptom, and cognitive function during follow-up stratified by social activity among the combined population. 32](#_Toc207380655)

[**Figure S6.** Estimated trajectories of physical disability, depressive symptom, and cognitive function during follow-up by cardiometabolic multimorbidity and social activity across different cohorts. 33](#_Toc207380656)

[**Figure S7**. Sensitivity analysis for estimated trajectories of physical disability, depressive symptoms, and cognitive function during follow-up by cardiometabolic multimorbidity and social activity by redefining social activity as respondents participated at least one social activities or groups in last month or year. 34](#_Toc207380657)

[**Figure S8.** Sensitivity analysis for estimated trajectories of physical disability, depressive symptoms, and cognitive function during follow-up by cardiometabolic multimorbidity and social activity by imputing missing covariates. 35](#_Toc207380658)

[**Figure S9.** Sensitivity analysis for estimated trajectories of physical disability, depressive symptoms, and cognitive function during follow-up by cardiometabolic multimorbidity and social activity by deleting participants with missing covariates. 36](#_Toc207380659)

[**Figure S10.** Random effect meta-analyses for the association of CMM with the joint trajectories of physical disability, depressive symptom and cognitive function. 37](#_Toc207380660)

# Supplementary Tables

## **Table S1.** Countries, waves, time periods and sample sizes included in present analyses according to studies.

| **Study** | **Country** | **Wave** | **Time period** | **Sample size** | |
| --- | --- | --- | --- | --- | --- |
|  |  |  |  | **Longitudinal changes of physical, psychological, and cognitive functions over time** | **Joint trajectories of physical, psychological, and cognitive functions** |
| CHARLS | China | 1-5 | 2011-2020 | 13667 | 9545 |
| ELSA | UK | 5-9 | 2010-2019 | 8205 | 6640 |
| HRS | US | 11-15 | 2012-2021 | 3368 | 1830 |
| SHARE | Austria | 5-9 | 2013-2022 | 2848 | 1028 |
|  | Germany | 5-9 | 2013-2022 | 4904 | 2822 |
|  | Sweden | 5-9 | 2013-2022 | 3583 | 2181 |
|  | Netherlands | 5-9 | 2013-2022 | 2917 | 468 |
|  | Spain | 5-9 | 2013-2022 | 4655 | 1255 |
|  | Italy | 5-9 | 2013-2022 | 3651 | 1921 |
|  | France | 5-9 | 2013-2022 | 3066 | 1667 |
|  | Denmark | 5-9 | 2013-2022 | 3309 | 2102 |
|  | Switzerland | 5-9 | 2013-2022 | 2399 | 1405 |
|  | Belgium | 5-9 | 2013-2022 | 4154 | 2130 |
|  | Israel | 5-9 | 2013-2022 | 1652 | 287 |
|  | Czech Republic | 5-9 | 2013-2022 | 4002 | 1973 |
|  | Luxembourg | 5-9 | 2013-2022 | 1417 | 608 |
|  | Slovenia | 5-9 | 2013-2022 | 2225 | 1236 |
|  | Estonia | 5-9 | 2013-2022 | 3756 | 1785 |

CHARLS, China Health and Retirement Longitudinal Study; HRS, US Health and Retirement Study; ELSA, English Longitudinal Study on Ageing; SHARE, Survey of Health, Ageing and Retirement in Europe.

## **Table S2.** Baseline characteristics of participants stratified by whether included in the analysis of joint trajectories.

|  | **Total**  **(N=73778)** | **Whether included in the analysis of joint trajectories** | | **P value** |
| --- | --- | --- | --- | --- |
|  |  | **Not included (N=32895)** | **Included**  **(N=40883)** |  |
| **Study** |  |  |  | <0.001 |
| CHARLS | 13667 (18.5) | 4122 (12.5) | 9545 (23.3) |  |
| ELSA | 8205 (11.1) | 1565 (4.8) | 6640 (16.2) |  |
| HRS | 3368 (4.6) | 1538 (4.7) | 1830 (4.5) |  |
| SHARE | 48538 (65.8) | 25670 (78.0) | 22868 (55.9) |  |
| **Age at baseline, mean (SD)** | 63.4 (9.1) | 64.0 (9.3) | 62.9 (8.9) | <0.001 |
| **Sex, n (%)** |  |  |  | 0.003 |
| Male | 32437 (44.0) | 14265 (43.4) | 18172 (44.4) |  |
| Female | 41341 (56.0) | 18630 (56.6) | 22711 (55.6) |  |
| **Educational level, n (%)** |  |  |  | <0.001 |
| Primary | 20005 (27.1) | 9187 (27.9) | 10818 (26.5) |  |
| Secondary | 37390 (50.7) | 16521 (50.2) | 20869 (51.0) |  |
| Tertiary | 15743 (21.3) | 7043 (21.4) | 8700 (21.3) |  |
| Unknown | 640 (0.9) | 144 (0.4) | 496 (1.2) |  |
| **Total household wealth, n (%)** |  |  |  | <0.001 |
| Q1 (lowest) | 17448 (23.6) | 8622 (26.2) | 8826 (21.6) |  |
| Q2 | 17460 (23.7) | 8184 (24.9) | 9276 (22.7) |  |
| Q3 | 17450 (23.7) | 7674 (23.3) | 9776 (23.9) |  |
| Q4 (highest) | 17451 (23.7) | 7120 (21.6) | 10331 (25.3) |  |
| Unknown | 3969 (5.4) | 1295 (3.9) | 2674 (6.5) |  |
| **Marital status, n (%)** |  |  |  | <0.001 |
| Married or partnered | 58351 (79.1) | 25788 (78.4) | 32563 (79.6) |  |
| Widowed | 7508 (10.2) | 3467 (10.5) | 4041 (9.9) |  |
| Separated or divorced or single | 7866 (10.7) | 3602 (10.9) | 4264 (10.4) |  |
| Unknown | 53 (0.1) | 38 (0.1) | 15 (0.0) |  |
| **Body mass index, kg/m^2^, n (%)** |  |  |  | 0.056 |
| <18.5 | 1259 (1.7) | 588 (1.8) | 671 (1.6) |  |
| 18.5-24.9 | 27275 (37.0) | 12011 (36.5) | 15264 (37.3) |  |
| 25-29.9 | 27092 (36.7) | 12178 (37.0) | 14914 (36.5) |  |
| ≥30 | 14081 (19.1) | 6258 (19.0) | 7823 (19.1) |  |
| Unknown | 4071 (5.5) | 1860 (5.7) | 2211 (5.4) |  |
| **Current smoking status, n (%)** |  |  |  | 0.582 |
| No | 59379 (80.5) | 26451 (80.4) | 32928 (80.5) |  |
| Yes | 14371 (19.5) | 6429 (19.5) | 7942 (19.4) |  |
| Unknown | 28 (0.0) | 15 (0.0) | 13 (0.0) |  |
| **Alcohol consumption, n (%)** |  |  |  | <0.001 |
| Less than weekly drinking | 40210 (54.5) | 18580 (56.5) | 21630 (52.9) |  |
| Weekly drinking or more | 32631 (44.2) | 14040 (42.7) | 18591 (45.5) |  |
| Unknown | 937 (1.3) | 275 (0.8) | 662 (1.6) |  |
| **Physical activity, n (%)** |  |  |  | <0.001 |
| Physical inactive | 14822 (20.1) | 5728 (17.4) | 9094 (22.2) |  |
| Physical active | 58953 (79.9) | 27164 (82.6) | 31789 (77.8) |  |
| Unknown | 3 (0.0) | 3 (0.0) | 0 (0.0) |  |
| **Baseline comorbidities, n (%)** |  |  |  |  |
| Hypertension | 29590 (40.1) | 13701 (41.7) | 15889 (38.9) | <0.001 |
| Cancer | 5190 (7.0) | 2319 (7.0) | 2871 (7.0) | 0.733 |
| Lung disease | 5597 (7.6) | 2678 (8.1) | 2919 (7.1) | <0.001 |
| **Social activity, n (%)** |  |  |  | <0.001 |
| Socially inactive | 19118 (46.8) | 38300 (51.9) | 19182 (58.3) |  |
| Socially active | 21765 (53.2) | 35478 (48.1) | 13713 (41.7) |  |
| **Cardiometabolic multimorbidity, n (%)** |  |  |  | <0.001 |
| No cardiometabolic disease | 55823 (75.7) | 24403 (74.2) | 31420 (76.9) |  |
| Single cardiometabolic disease | 14784 (20.0) | 6847 (20.8) | 7937 (19.4) |  |
| Cardiometabolic multimorbidity | 3171 (4.3) | 1645 (5.0) | 1526 (3.7) |  |

## **Table S3.** Harmonized strategies for key variables included in the study.

| **Variables** | **Harmonized values** | **CHARLS** | **HRS** | **ELSA** | | | **SHARE** |
| --- | --- | --- | --- | --- | --- | --- | --- |
| **Diabetes** | Yes | Self-report of diabetes or high blood sugar | | | | | |
|  | No | Otherwise |  |  | | |  |
| **Heart disease** | Yes | Self-report of heart attack, coronary heart disease, angina, congestive heart failure, or other heart problems | Self-report of heart attack, coronary heart disease, angina, congestive heart failure, or other heart problems | Self-report of angina, a heart attack (including myocardial infarction or coronary thrombosis), congestive heart failure, a heart murmur, an abnormal heart rhythm, or any other heart trouble | | | Self-report of heart attack, including myocardial infarction or coronary thrombosis, or any other heart problem, including congestive heart failure |
|  | No | Otherwise | | | | | |
| **Stroke** | Yes | Self-report of stroke | Self-report of stroke or transient ischemic attack | Self-report of stroke or cerebrovascular disease | | | Self-report of stroke or cerebrovascular disease |
|  | No | Otherwise | | | | | |
| **Social activity** | Yes | Have you done the following social activities in the last month:  Interacted with friends  Played Ma-jong, played chess, played cards, or went to community club;  Provided help to family, friends, or neighbours who do not live with you and who did not pay you for the help;  Went to a sport, social, or other kind of club;  Took part in a community-related organisation;  Done voluntary or charity work;  Cared for a sick or disabled adult who does not live with you and who did not pay you for the help;  Attended an educational or training course.  Those reported doing one of the aforementioned activities in the last month were considered to be socially active. | How often you do each active:  Care for a sick or disabled adult;  Do activities with grandchildren, nieces/nephews, or neighbourhood children;  Do volunteer work with children or young people;  Do any other volunteer or charity work;  Attend an educational or training course;  Go to a sport, social, or other club;  Attend meetings of non-religious organisations, such as political, community, or other interest groups.  Those reported doing one of the aforementioned activities at least once a month were considered to be socially active. | Being a member of the following organisations, clubs or societies:  Political party, trade union or environmental groups;  Tenants groups, resident groups, Neighbourhood watch;  Charitable associations;  Education, arts or  music groups or evening classes;  Social Clubs;  Sports clubs, gyms, exercise classes;  Any other organisations, clubs or societies. | | | Have you done the following social activities in the past twelve months, and how often in the past twelve months:  Done voluntary or charity work;  Attended an educational or training course;  Gone to a sport, social or other kind of club;  Taken part in a political or community-related organisation.  Those reported doing one of the aforementioned activities at least once a month were considered to be socially active. |
|  | No | Otherwise | Otherwise | Otherwise | | | Otherwise |
| **Physical disability** |  | Z-score of difficulties performing activities of daily living (ADL), including dressing, bathing and showering, eating, getting in and out of bed, using toilet, and controlling urination and defecation. | Z-score of difficulties performing activities of daily living (ADL), including walking across a room, dressing, bathing and showering, eating, getting in and out of bed, and using toilet. | | | | |
| **Depressive symptom** |  | Z-score of CESD-10 | Z-score of CESD-8 | | | | Z-score of EURO-D |
| **Cognitive function** |  | Z-score of the summed score for the following domains: immediate word recall, delayed word recall, serial 7’ subtraction test, orientation, and picture drawing task. | Z-score of the summed score for the following domains: immediate word recall, delayed word recall, and serial 7’ subtraction test, orientation. | Z-score of the summed score for the following domains: immediate word recall, delayed word recall, and orientation. | | | Z-score of the summed score for the following domains: immediate word recall, delayed word recall, serial 7’ subtraction test, animal fluency test and orientation. |
| **Educational level** | Primary | Less than upper secondary education | | | | | |
|  | Secondary | Upper secondary & vocational training | | | | | |
|  | Tertiary | Tertiary education | | | | | |
| **Total household wealth** | Q1 (lowest) | The sum of all wealth components (including residence, vehicles, saving accounts, etc.) minus other debts at the couple level (the respondent and spouse, if any) in local currencies. | | | | | |
|  | Q2 |  |  |  |  |  |  |
|  | Q3 |  |  |  |  |  |  |
|  | Q4 (highest) |  |  |  |  |  |  |
| **Body mass index** |  | Assessed at wave 1 | Assessed at wave 11 | | Body mass index was not available in wave 5, therefore, the mean values of BMI in wave 4 and wave 6 was calculated to represent baseline body mass index. | Assessed at wave 5 | |
| **Smoking status** | Yes | Smokes at the present time | | | | | |
|  | No | Does not smoke at the present time | | | | | |
| **Alcohol consumption** | Weekly drinking or more | The frequency of drinking behaviour during the last year: Once a week/2 to 3 days a week/4 to 6 days a week/Daily/ Twice a day / More than twice a day | The frequency of drinking:1-7 days/week | | | | Whether drinks weekly or has had an alcoholic drink during the last 7 days: Yes |
|  | Less than weekly drinking | None or doesn’t drink / Once a month / 2 to 3 days a month | 0 day/week | | | | No |
| **Physical activity** | Yes | The number of days of vigorous/moderate physical activity for at least 10 minutes every week: 1-7 days | Frequency of taking part in vigorous/moderate physical activity: everyday / more than once a week / once a week / one to three times a month | | | | |
|  | No | None | Hardly ever or never taking part in vigorous/moderate physical activity | | | | |

## **Table S4.** Joint trajectory models’ results of model fitting process.

| **Number of classes** | **Trajectory shapes** | **BIC** | **Group membership** | | | | | **Ave PP** | | | | |
| --- | --- | --- | --- | --- | --- | --- | --- | --- | --- | --- | --- | --- |
| 2 | (1 1), (1 1), (1 1) | -612499.0 | 6.8 | 93.2 |  |  |  | 96.5 | 99.7 |  |  |  |
| 2 | (2 2), (2 2), (2 2) | -612189.3 | 6.9 | 93.1 |  |  |  | 96.3 | 99.8 |  |  |  |
| 2 | (3 3), (3 3), (3 3) | -612111.5 | 6.9 | 93.1 |  |  |  | 96.1 | 99.8 |  |  |  |
| 3 | (1 1 1), (1 1 1), (1 1 1) | -588627.8 | 5.0 | 37.3 | 57.2 |  |  | 96.4 | 89.2 | 92.3 |  |  |
| 3 | (2 2 2), (2 2 2), (2 2 2) | -588296.4 | 5.6 | 37.2 | 57.3 |  |  | 96.1 | 89.2 | 92.4 |  |  |
| 3 | (3 3 3), (3 3 3), (3 3 3) | -588181.0 | 5.6 | 37.2 | 57.2 |  |  | 96.1 | 89.1 | 92.5 |  |  |
| 4 | (1 1 1 1), (1 1 1 1), (1 1 1 1) | -574317.3 | 4.7 | 14.6 | 32.6 | 48.1 |  | 96.4 | 86.2 | 84.6 | 90.3 |  |
| 4 | (2 2 2 2), (2 2 2 2), (2 2 2 2) | -574014.2 | 4.8 | 14.5 | 32.6 | 48.1 |  | 96.3 | 86.2 | 84.4 | 90.3 |  |
| **4** | **(3 3 3 3), (3 3 3 3), (3 3 3 3)** | **-573907.0** | **4.7** | **14.6** | **32.6** | **48.1** |  | **97.2** | **86.2** | **84.4** | **90.3** |  |
| 5 | (1 1 1 1 1), (1 1 1 1 1), (1 1 1 1 1) | -568442.5 | 4.6 | 12.7 | 14.5 | 43.7 | 24.5 | 96.3 | 84.5 | 81.7 | 84.1 | 83.6 |
| 5 | (2 2 2 2 2), (2 2 2 2 2), (2 2 2 2 2) | -568132.2 | 4.6 | 12.7 | 14.5 | 43.6 | 24.6 | 96.2 | 84.7 | 81.7 | 84.1 | 83.7 |
| 5 | (3 3 3 3 3), (3 3 3 3 3), (3 3 3 3 3) | -568016.6 | 4.6 | 12.6 | 14.5 | 43.6 | 24.7 | 96.3 | 84.7 | 82.0 | 84.0 | 83.9 |

**Abbreviations:** **BIC**, Bayesian Information Criterion; **Ave PP**, Average posterior probability assignment

## **Table S5.** Baseline characteristics of participants included in the analysis of longitudinal changes of physical disability, depressive symptom, and cognitive function by baseline cardiometabolic diseases.

|  | **Total**  **(N=73778)** | **Cardiometabolic status at baseline** | | | **P value** |
| --- | --- | --- | --- | --- | --- |
|  |  | **No CMD**  **(N=55823)** | **Single CMD**  **(N=14784)** | **CMM**  **(N=3171)** |  |
| **Study** |  |  |  |  | <0.001 |
| CHARLS | 13667 (18.5) | 11178 (20.0) | 2154 (14.6) | 335 (10.6) |  |
| ELSA | 8205 (11.1) | 6041 (10.8) | 1864 (12.6) | 300 (9.5) |  |
| HRS | 3368 (4.6) | 1921 (3.4) | 1107 (7.5) | 340 (10.7) |  |
| SHARE | 48538 (65.8) | 36683 (65.7) | 9659 (65.3) | 2196 (69.3) |  |
| **Age at baseline, mean (SD)** | 63.4 (9.1) | 62.3 (8.9) | 66.6 (8.9) | 68.7 (8.5) | <0.001 |
| **Sex, n (%)** |  |  |  |  | <0.001 |
| Male | 32437 (44.0) | 23780 (42.6) | 7105 (48.1) | 1552 (48.9) |  |
| Female | 41341 (56.0) | 32043 (57.4) | 7679 (51.9) | 1619 (51.1) |  |
| **Educational level, n (%)** |  |  |  |  | <0.001 |
| Primary | 20005 (27.1) | 14762 (26.4) | 4246 (28.7) | 997 (31.4) |  |
| Secondary | 37390 (50.7) | 28281 (50.7) | 7495 (50.7) | 1614 (50.9) |  |
| Tertiary | 15743 (21.3) | 12333 (22.1) | 2873 (19.4) | 537 (16.9) |  |
| Unknown | 640 (0.9) | 447 (0.8) | 170 (1.1) | 23 (0.7) |  |
| **Total household wealth, n (%)** |  |  |  |  | <0.001 |
| Q1 (lowest) | 17448 (23.6) | 11927 (21.4) | 4345 (29.4) | 1176 (37.1) |  |
| Q2 | 17460 (23.7) | 12950 (23.2) | 3685 (24.9) | 825 (26.0) |  |
| Q3 | 17450 (23.7) | 13604 (24.4) | 3257 (22.0) | 589 (18.6) |  |
| Q4 (highest) | 17451 (23.7) | 14087 (25.2) | 2876 (19.5) | 488 (15.4) |  |
| Unknown | 3969 (5.4) | 3255 (5.8) | 621 (4.2) | 93 (2.9) |  |
| **Marital status, n (%)** |  |  |  |  | <0.001 |
| Married or partnered | 58351 (79.1) | 44962 (80.5) | 11134 (75.3) | 2255 (71.1) |  |
| Widowed | 7508 (10.2) | 4942 (8.9) | 2028 (13.7) | 538 (17.0) |  |
| Separated or divorced or single | 7866 (10.7) | 5885 (10.5) | 1610 (10.9) | 371 (11.7) |  |
| Unknown | 53 (0.1) | 34 (0.1) | 12 (0.1) | 7 (0.2) |  |
| **Body mass index, n (%)** |  |  |  |  | <0.001 |
| <18.5 kg/m^2^ | 1259 (1.7) | 1055 (1.9) | 187 (1.3) | 17 (0.5) |  |
| 18.5-24.9 kg/m^2^ | 27275 (37.0) | 22777 (40.8) | 3891 (26.3) | 607 (19.1) |  |
| 25-29.9 kg/m^2^ | 27092 (36.7) | 20137 (36.1) | 5801 (39.2) | 1154 (36.4) |  |
| ≥30 kg/m^2^ | 14081 (19.1) | 8769 (15.7) | 4098 (27.7) | 1214 (38.3) |  |
| Unknown | 4071 (5.5) | 3085 (5.5) | 807 (5.5) | 179 (5.6) |  |
| **Current smoking status, n (%)** |  |  |  |  | <0.001 |
| No | 59379 (80.5) | 44315 (79.4) | 12403 (83.9) | 2661 (83.9) |  |
| Yes | 14371 (19.5) | 11488 (20.6) | 2376 (16.1) | 507 (16.0) |  |
| Unknown | 28 (0.0) | 20 (0.0) | 5 (0.0) | 3 (0.1) |  |
| **Alcohol consumption, n (%)** |  |  |  |  | <0.001 |
| Less than weekly drinking | 40210 (54.5) | 29201 (52.3) | 8828 (59.7) | 2181 (68.8) |  |
| Weekly drinking or more | 32631 (44.2) | 25845 (46.3) | 5826 (39.4) | 960 (30.3) |  |
| Unknown | 937 (1.3) | 777 (1.4) | 130 (0.9) | 30 (0.9) |  |
| **Physical activity, n (%)** |  |  |  |  | <0.001 |
| Physical inactive | 14822 (20.1) | 10654 (19.1) | 3272 (22.1) | 896 (28.3) |  |
| Physical active | 58953 (79.9) | 45167 (80.9) | 11511 (77.9) | 2275 (71.7) |  |
| Unknown | 3 (0.0) | 2 (0.0) | 1 (0.0) | 0 (0.0) |  |
| **Baseline comorbidities, n (%)** |  |  |  |  |  |
| Hypertension | 29590 (40.1) | 18325 (32.8) | 8791 (59.5) | 2474 (78.0) | <0.001 |
| Cancer | 5190 (7.0) | 3446 (6.2) | 1354 (9.2) | 390 (12.3) | <0.001 |
| Lung disease | 5597 (7.6) | 3478 (6.2) | 1573 (10.6) | 546 (17.2) | <0.001 |
| **Social activity, n (%)** |  |  |  |  | <0.001 |
| Socially inactive | 38300 (51.9) | 28289 (50.7) | 8071 (54.6) | 1940 (61.2) |  |
| Socially active | 35478 (48.1) | 27534 (49.3) | 6713 (45.4) | 1231 (38.8) |  |

CMD: cardiometabolic diseases; CMM: cardiometabolic multimorbidity.

## **Table S6.** Baseline characteristics of participants included in the analysis of joint trajectories of physical disability, depressive symptom, and cognitive function by baseline cardiometabolic diseases.

|  | **Total**  **(N=40883)** | **Cardiometabolic status at baseline** | | | **P value** |
| --- | --- | --- | --- | --- | --- |
|  |  | **No CMD**  **(N=31420)** | **Single CMD**  **(N=7937)** | **CMM**  **(N=1526)** |  |
| **Study** |  |  |  |  | <0.001 |
| CHARLS | 9545 (23.3) | 7859 (25.0) | 1485 (18.7) | 201 (13.2) |  |
| ELSA | 6640 (16.2) | 5030 (16.0) | 1407 (17.7) | 203 (13.3) |  |
| HRS | 1830 (4.5) | 937 (3.0) | 657 (8.3) | 236 (15.5) |  |
| SHARE | 22868 (55.9) | 17594 (56.0) | 4388 (55.3) | 886 (58.1) |  |
| **Age at baseline, mean (SD)** | 62.9 (8.9) | 61.9 (8.7) | 66.0 (8.8) | 68.3 (8.1) | <0.001 |
| **Sex, n (%)** |  |  |  |  | <0.001 |
| Male | 18172 (44.4) | 13677 (43.5) | 3749 (47.2) | 746 (48.9) |  |
| Female | 22711 (55.6) | 17743 (56.5) | 4188 (52.8) | 780 (51.1) |  |
| **Educational level, n (%)** |  |  |  |  | <0.001 |
| Primary | 10818 (26.5) | 8187 (26.1) | 2180 (27.5) | 451 (29.6) |  |
| Secondary | 20869 (51.0) | 16055 (51.1) | 4036 (50.9) | 778 (51.0) |  |
| Tertiary | 8700 (21.3) | 6820 (21.7) | 1599 (20.1) | 281 (18.4) |  |
| Unknown | 496 (1.2) | 358 (1.1) | 122 (1.5) | 16 (1.0) |  |
| **Total household wealth, n (%)** |  |  |  |  | <0.001 |
| Q1 (lowest) | 8826 (21.6) | 6149 (19.6) | 2163 (27.3) | 514 (33.7) |  |
| Q2 | 9276 (22.7) | 6947 (22.1) | 1931 (24.3) | 398 (26.1) |  |
| Q3 | 9776 (23.9) | 7676 (24.4) | 1798 (22.7) | 302 (19.8) |  |
| Q4 (highest) | 10331 (25.3) | 8440 (26.9) | 1633 (20.6) | 258 (16.9) |  |
| Unknown | 2674 (6.5) | 2208 (7.0) | 412 (5.2) | 54 (3.5) |  |
| **Marital status, n (%)** |  |  |  |  | <0.001 |
| Married or partnered | 32563 (79.6) | 25467 (81.1) | 6005 (75.7) | 1091 (71.5) |  |
| Widowed | 4041 (9.9) | 2717 (8.6) | 1078 (13.6) | 246 (16.1) |  |
| Separated or divorced or single | 4264 (10.4) | 3224 (10.3) | 853 (10.7) | 187 (12.3) |  |
| Unknown | 15 (0.0) | 12 (0.0) | 1 (0.0) | 2 (0.1) |  |
| **Body mass index, kg/m^2^, n (%)** |  |  |  |  | <0.001 |
| <18.5 | 671 (1.6) | 580 (1.8) | 84 (1.1) | 7 (0.5) |  |
| 18.5-24.9 | 15264 (37.3) | 12916 (41.1) | 2071 (26.1) | 277 (18.2) |  |
| 25-29.9 | 14914 (36.5) | 11270 (35.9) | 3088 (38.9) | 556 (36.4) |  |
| ≥30 | 7823 (19.1) | 4925 (15.7) | 2288 (28.8) | 610 (40.0) |  |
| Unknown | 2211 (5.4) | 1729 (5.5) | 406 (5.1) | 76 (5.0) |  |
| **Current smoking status, n (%)** |  |  |  |  | <0.001 |
| No | 32928 (80.5) | 24937 (79.4) | 6702 (84.4) | 1289 (84.5) |  |
| Yes | 7942 (19.4) | 6475 (20.6) | 1232 (15.5) | 235 (15.4) |  |
| Unknown | 13 (0.0) | 8 (0.0) | 3 (0.0) | 2 (0.1) |  |
| **Alcohol consumption, n (%)** |  |  |  |  | <0.001 |
| Less than weekly drinking | 21630 (52.9) | 15986 (50.9) | 4619 (58.2) | 1025 (67.2) |  |
| Weekly drinking or more | 18591 (45.5) | 14875 (47.3) | 3226 (40.6) | 490 (32.1) |  |
| Unknown | 662 (1.6) | 559 (1.8) | 92 (1.2) | 11 (0.7) |  |
| **Physical activity, n (%)** |  |  |  |  | <0.001 |
| Physical inactive | 9094 (22.2) | 6807 (21.7) | 1849 (23.3) | 438 (28.7) |  |
| Physical active | 31789 (77.8) | 24613 (78.3) | 6088 (76.7) | 1088 (71.3) |  |
| **Baseline comorbidities, n (%)** |  |  |  |  |  |
| Hypertension | 15889 (38.9) | 10007 (31.8) | 4705 (59.3) | 1177 (77.1) | <0.001 |
| Cancer | 2871 (7.0) | 1935 (6.2) | 749 (9.4) | 187 (12.3) | <0.001 |
| Lung disease | 2919 (7.1) | 1891 (6.0) | 783 (9.9) | 245 (16.1) | <0.001 |
| **Social activity, n (%)** |  |  |  |  | <0.001 |
| Socially inactive | 19118 (46.8) | 14471 (46.1) | 3836 (48.3) | 811 (53.1) |  |
| Socially active | 21765 (53.2) | 16949 (53.9) | 4101 (51.7) | 715 (46.9) |  |

CMD: cardiometabolic diseases; CMM: cardiometabolic multimorbidity.

## **Table S7.** Baseline characteristics of participants by identified joint trajectories of physical disability, depressive symptom, and cognitive function.

|  | **Total**  **(N=40883)** | **Identified joint trajectories** | | | | **P value** |
| --- | --- | --- | --- | --- | --- | --- |
|  |  | **Favourable trajectories of physical disability, depressive symptom and cognitive function (N=19680)** | **Favourable trajectories of physical disability and depressive symptom, and worsening cognitive function (N=13330)** | **Favourable trajectory of physical disability, and worsening depressive symptom and cognitive function (N=5949)** | **Rapidly-worsening physical disability and worsening depressive symptom and cognitive function(N=1924)** |  |
| **Study** |  |  |  |  |  | <0.001 |
| CHARLS | 9545 (23.3) | 3827 (19.4) | 3518 (26.4) | 1598 (26.9) | 602 (31.3) |  |
| ELSA | 6640 (16.2) | 3117 (15.8) | 2290 (17.2) | 737 (12.4) | 496 (25.8) |  |
| HRS | 1830 (4.5) | 933 (4.7) | 532 (4.0) | 250 (4.2) | 115 (6.0) |  |
| SHARE | 22868 (55.9) | 11803 (60.0) | 6990 (52.4) | 3364 (56.5) | 711 (37.0) |  |
| **Age at baseline, mean (SD)** | 62.9 (8.9) | 60.9 (8.0) | 65.2 (9.2) | 63.3 (9.3) | 66.6 (9.4) | <0.001 |
| **Sex, n (%)** |  |  |  |  |  | <0.001 |
| Male | 18172 (44.4) | 8769 (44.6) | 6851 (51.4) | 1741 (29.3) | 811 (42.2) |  |
| Female | 22711 (55.6) | 10911 (55.4) | 6479 (48.6) | 4208 (70.7) | 1113 (57.8) |  |
| **Educational level, n (%)** |  |  |  |  |  | <0.001 |
| Primary | 10818 (26.5) | 2261 (11.5) | 5355 (40.2) | 2347 (39.5) | 855 (44.4) |  |
| Secondary | 20869 (51.0) | 11047 (56.1) | 6220 (46.7) | 2800 (47.1) | 802 (41.7) |  |
| Tertiary | 8700 (21.3) | 6179 (31.4) | 1555 (11.7) | 741 (12.5) | 225 (11.7) |  |
| Unknown | 496 (1.2) | 193 (1.0) | 200 (1.5) | 61 (1.0) | 42 (2.2) |  |
| **Total household wealth, n (%)** |  |  |  |  |  | <0.001 |
| Q1 (lowest) | 8826 (21.6) | 3221 (16.4) | 3018 (22.6) | 1855 (31.2) | 732 (38.0) |  |
| Q2 | 9276 (22.7) | 4102 (20.8) | 3250 (24.4) | 1473 (24.8) | 451 (23.4) |  |
| Q3 | 9776 (23.9) | 4889 (24.8) | 3234 (24.3) | 1282 (21.5) | 371 (19.3) |  |
| Q4 (highest) | 10331 (25.3) | 6276 (31.9) | 2876 (21.6) | 966 (16.2) | 213 (11.1) |  |
| Unknown | 2674 (6.5) | 1192 (6.1) | 952 (7.1) | 373 (6.3) | 157 (8.2) |  |
| **Marital status, n (%)** |  |  |  |  |  | <0.001 |
| Married or partnered | 32563 (79.6) | 16355 (83.1) | 10500 (78.8) | 4352 (73.2) | 1356 (70.5) |  |
| Widowed | 4041 (9.9) | 1294 (6.6) | 1605 (12.0) | 821 (13.8) | 321 (16.7) |  |
| Separated or divorced or single | 4264 (10.4) | 2026 (10.3) | 1217 (9.1) | 775 (13.0) | 246 (12.8) |  |
| Unknown | 15 (0.0) | 5 (0.0) | 8 (0.1) | 1 (0.0) | 1 (0.1) |  |
| **Body mass index, kg/m^2^, n (%)** |  |  |  |  |  | <0.001 |
| <18.5 | 671 (1.6) | 242 (1.2) | 230 (1.7) | 149 (2.5) | 50 (2.6) |  |
| 18.5-24.9 | 15264 (37.3) | 7558 (38.4) | 5029 (37.7) | 2109 (35.5) | 568 (29.5) |  |
| 25-29.9 | 14914 (36.5) | 7330 (37.2) | 5009 (37.6) | 2032 (34.2) | 543 (28.2) |  |
| ≥30 | 7823 (19.1) | 3475 (17.7) | 2415 (18.1) | 1343 (22.6) | 590 (30.7) |  |
| Unknown | 2211 (5.4) | 1075 (5.5) | 647 (4.9) | 316 (5.3) | 173 (9.0) |  |
| **Current smoking status, n (%)** |  |  |  |  |  | <0.001 |
| No | 32928 (80.5) | 16133 (82.0) | 10599 (79.5) | 4694 (78.9) | 1502 (78.1) |  |
| Yes | 7942 (19.4) | 3541 (18.0) | 2725 (20.4) | 1254 (21.1) | 422 (21.9) |  |
| Unknown | 13 (0.0) | 6 (0.0) | 6 (0.0) | 1 (0.0) | 0 (0.0) |  |
| **Alcohol consumption, n (%)** |  |  |  |  |  | <0.001 |
| Less than weekly drinking | 21630 (52.9) | 9127 (46.4) | 7336 (55.0) | 3871 (65.1) | 1296 (67.4) |  |
| Weekly drinking or more | 18591 (45.5) | 10293 (52.3) | 5730 (43.0) | 1984 (33.4) | 584 (30.4) |  |
| Unknown | 662 (1.6) | 260 (1.3) | 264 (2.0) | 94 (1.6) | 44 (2.3) |  |
| **Physical activity, n (%)** |  |  |  |  |  | <0.001 |
| Physical inactive | 9094 (22.2) | 3262 (16.6) | 3237 (24.3) | 1639 (27.6) | 956 (49.7) |  |
| Physical active | 31789 (77.8) | 16418 (83.4) | 10093 (75.7) | 4310 (72.4) | 968 (50.3) |  |
| **Baseline comorbidities, n (%)** |  |  |  |  |  |  |
| Hypertension | 15889 (38.9) | 6758 (34.3) | 5387 (40.4) | 2676 (45.0) | 1068 (55.5) | <0.001 |
| Cancer | 2871 (7.0) | 1318 (6.7) | 890 (6.7) | 492 (8.3) | 171 (8.9) | <0.001 |
| Lung disease | 2919 (7.1) | 970 (4.9) | 898 (6.7) | 741 (12.5) | 310 (16.1) | <0.001 |
| **Social activity, n (%)** |  |  |  |  |  | <0.001 |
| Socially inactive | 19118 (46.8) | 7530 (38.3) | 6970 (52.3) | 3483 (58.5) | 1135 (59.0) |  |
| Socially active | 21765 (53.2) | 12150 (61.7) | 6360 (47.7) | 2466 (41.5) | 789 (41.0) |  |
| **Cardiometabolic multimorbidity, n (%)** | |  |  |  |  | <0.001 |
| No cardiometabolic disease | 31420 (76.9) | 16203 (82.3) | 10131 (76.0) | 4034 (67.8) | 1052 (54.7) |  |
| Single cardiometabolic disease | 7937 (19.4) | 3058 (15.5) | 2681 (20.1) | 1536 (25.8) | 662 (34.4) |  |
| Cardiometabolic multimorbidity | 1526 (3.7) | 419 (2.1) | 518 (3.9) | 379 (6.4) | 210 (10.9) |  |

## **Table S8.** Addictive and multiplicative estimates of the modification effect of social activity for the association between CMM and joint trajectories of physical disability, depressive symptom, and cognitive function.

|  | **Multiplicative interaction** | | **Additive interaction** | |
| --- | --- | --- | --- | --- |
|  | **ROR (95% CI)** | **P value** | **RERI (95% CI)** | **P value** |
| **Single CMD and social inactivity** |  |  |  |  |
| Favourable trajectories of physical disability and depressive symptom, and worsening cognitive function | 0.98 (0.88-1.11) | 0.797 | **0.28 (0.05-0.50)** | **0.008** |
| Favourable trajectory of physical disability, and worsening depressive symptom and cognitive function | 0.95 (0.82-1.09) | 0.462 | **1.15 (0.70-1.60)** | **<0.001** |
| Rapidly-worsening physical disability and worsening depressive symptom and cognitive function | 0.88 (0.71-1.09) | 0.249 | **2.59 (1.58-3.61)** | **<0.001** |
| **CMM and social inactivity** |  |  |  |  |
| Favourable trajectories of physical disability and depressive symptom, and worsening cognitive function | 1.14 (0.88-1.49) | 0.322 | **1.10 (0.32-1.89)** | **0.003** |
| Favourable trajectory of physical disability, and worsening depressive symptom and cognitive function | 1.05 (0.79-1.41) | 0.727 | **3.69 (1.83-5.55)** | **<0.001** |
| Rapidly-worsening physical disability and worsening depressive symptom and cognitive function | 1.08 (0.75-1.55) | 0.692 | **10.20 (5.37-15.04)** | **<0.001** |

The favourable trajectories of physical disability, depressive symptom, and cognitive function was the reference group. P values are corrected with Benjamini–Hochberg procedure.

RERI: relative excess risk due to interaction (additive interaction estimates); ROR: ratio of odds ratio (multiplicative interaction estimates).

## **Table S9.** Association of cardiometabolic multimorbidity and social activity with joint trajectories of physical disability, depressive symptom, and cognitive function stratified by sex.

|  | **Favourable trajectories of physical disability, depressive symptom and cognitive function** | **Favourable trajectories of physical disability and depressive symptom, and worsening cognitive function** | **Favourable trajectory of physical disability, and worsening depressive symptom and cognitive function** | **Rapidly-worsening physical disability and worsening depressive symptom and cognitive function** |
| --- | --- | --- | --- | --- |
| **Male** |  |  |  |  |
| **Baseline CMM status** |  |  |  |  |
| No CMD | 1.00 (reference) | 1.00 (reference) | 1.00 (reference) | 1.00 (reference) |
| Single CMD | 1.00 (reference) | 1.07 (0.98-1.18) | **1.58 (1.39-1.81)** | **1.93 (1.61-2.31)** |
| CMM | 1.00 (reference) | **1.35 (1.11-1.65)** | **2.74 (2.14-3.49)** | **3.39 (2.51-4.58)** |
| **CMM combined with social activity** | |  |  |  |
| No CMD & socially active | 1.00 (reference) | 1.00 (reference) | 1.00 (reference) | 1.00 (reference) |
| No CMD & socially inactive | 1.00 (reference) | **1.53 (1.41-1.66)** | **1.71 (1.50-1.96)** | **2.17 (1.77-2.67)** |
| Single CMD & socially active | 1.00 (reference) | 1.11 (0.98-1.25) | **1.62 (1.34-1.96)** | **2.28 (1.76-2.96)** |
| Single CMD & socially inactive | 1.00 (reference) | **1.57 (1.38-1.79)** | **2.62 (2.18-3.16)** | **3.61 (2.79-4.67)** |
| CMM & socially active | 1.00 (reference) | 1.14 (0.87-1.50) | **2.61 (1.84-3.70)** | **2.60 (1.61-4.18)** |
| CMM & socially inactive | 1.00 (reference) | **2.48 (1.86-3.29)** | **5.17 (3.68-7.27)** | **9.09 (6.09-13.6)** |
| **Female** |  |  |  |  |
| **Baseline CMM status** |  |  |  |  |
| No CMD | 1.00 (reference) | 1.00 (reference) | 1.00 (reference) | 1.00 (reference) |
| Single CMD | 1.00 (reference) | 1.08 (0.98-1.19) | **1.71 (1.55-1.89)** | **2.15 (1.84-2.52)** |
| CMM | 1.00 (reference) | **1.27 (1.02-1.57)** | **2.38 (1.93-2.95)** | **3.31 (2.51-4.36)** |
| **CMM combined with social activity** |  |  |  |  |
| No CMD & socially active | 1.00 (reference) | 1.00 (reference) | 1.00 (reference) | 1.00 (reference) |
| No CMD & socially inactive | 1.00 (reference) | **1.49 (1.38-1.61)** | **1.75 (1.60-1.92)** | **1.87 (1.56-2.23)** |
| Single CMD & socially active | 1.00 (reference) | 1.08 (0.95-1.23) | **1.77 (1.54-2.04)** | **2.04 (1.63-2.57)** |
| Single CMD & socially inactive | 1.00 (reference) | **1.60 (1.40-1.84)** | **2.91 (2.53-3.36)** | **4.16 (3.33-5.19)** |
| CMM & socially active | 1.00 (reference) | **1.38 (1.03-1.84)** | **2.40 (1.78-3.24)** | **3.65 (2.47-5.38)** |
| CMM & socially inactive | 1.00 (reference) | **1.72 (1.26-2.35)** | **4.04 (2.99-5.45)** | **5.58 (3.78-8.24)** |

Covariates include cohort, age at baseline, educational level, total household wealth, marital status, body mass index, current smoking status, alcohol consumption, physical activity, baseline hypertension, lung diseases, and cancer. For the model of baseline CMM, social activity was additionally adjusted.

Odds ratios and 95% confidence intervals were reported in the table. CMD: cardiometabolic disease; CMM: cardiometabolic multimorbidity.

## **Table S10.** Association of cardiometabolic multimorbidity and social activity with joint trajectories of physical disability, depressive symptom, and cognitive function stratified by age.

|  | **Favourable trajectories of physical disability, depressive symptom and cognitive function** | **Favourable trajectories of physical disability and depressive symptom, and worsening cognitive function** | **Favourable trajectory of physical disability, and worsening depressive symptom and cognitive function** | **Rapidly-worsening physical disability and worsening depressive symptom and cognitive function** |
| --- | --- | --- | --- | --- |
| **45-64 years** |  |  |  |  |
| **Baseline CMM status** |  |  |  |  |
| No CMD | 1.00 (reference) | 1.00 (reference) | 1.00 (reference) | 1.00 (reference) |
| Single CMD | 1.00 (reference) | **1.12 (1.02-1.24)** | **1.76 (1.58-1.96)** | **2.57 (2.15-3.06)** |
| CMM | 1.00 (reference) | **1.54 (1.21-1.97)** | **2.80 (2.17-3.62)** | **4.78 (3.40-6.72)** |
| **CMM combined with social activity** | |  |  |  |
| No CMD & socially active | 1.00 (reference) | 1.00 (reference) | 1.00 (reference) | 1.00 (reference) |
| No CMD & socially inactive | 1.00 (reference) | **1.54 (1.43-1.66)** | **1.74 (1.58-1.91)** | **1.75 (1.44-2.11)** |
| Single CMD & socially active | 1.00 (reference) | **1.15 (1.00-1.32)** | **1.91 (1.63-2.24)** | **2.81 (2.18-3.64)** |
| Single CMD & socially inactive | 1.00 (reference) | **1.68 (1.47-1.93)** | **2.83 (2.43-3.30)** | **4.12 (3.22-5.28)** |
| CMM & socially active | 1.00 (reference) | **1.31 (0.91-1.89)** | **2.70 (1.87-3.91)** | **3.97 (2.35-6.70)** |
| CMM & socially inactive | 1.00 (reference) | **2.74 (1.95-3.84)** | **5.17 (3.64-7.36)** | **9.73 (6.19-15.3)** |
| **65-74 years** |  |  |  |  |
| **Baseline CMM status** |  |  |  |  |
| No CMD | 1.00 (reference) | 1.00 (reference) | 1.00 (reference) | 1.00 (reference) |
| Single CMD | 1.00 (reference) | 1.07 (0.97-1.19) | **1.57 (1.38-1.80)** | **1.91 (1.56-2.33)** |
| CMM | 1.00 (reference) | **1.24 (1.00-1.53)** | **2.45 (1.92-3.12)** | **3.45 (2.51-4.73)** |
| **CMM combined with social activity** |  |  |  |  |
| No CMD & socially active | 1.00 (reference) | 1.00 (reference) | 1.00 (reference) | 1.00 (reference) |
| No CMD & socially inactive | 1.00 (reference) | **1.42 (1.28-1.57)** | **1.72 (1.48-1.99)** | **2.21 (1.74-2.82)** |
| Single CMD & socially active | 1.00 (reference) | 1.05 (0.91-1.20) | **1.59 (1.32-1.92)** | **1.95 (1.45-2.62)** |
| Single CMD & socially inactive | 1.00 (reference) | **1.57 (1.34-1.83)** | **2.70 (2.22-3.27)** | **4.19 (3.14-5.60)** |
| CMM & socially active | 1.00 (reference) | 1.16 (0.87-1.56) | **2.74 (1.97-3.81)** | **4.26 (2.73-6.63)** |
| CMM & socially inactive | 1.00 (reference) | **1.83 (1.34-2.49)** | **3.80 (2.68-5.37)** | **6.43 (4.11-10.1)** |
| **≥75 years** |  |  |  |  |
| **Baseline CMM status** |  |  |  |  |
| No CMD | 1.00 (reference) | 1.00 (reference) | 1.00 (reference) | 1.00 (reference) |
| Single CMD | 1.00 (reference) | 1.05 (0.88-1.25) | **1.52 (1.22-1.89)** | **1.71 (1.31-2.24)** |
| CMM | 1.00 (reference) | **1.43 (1.00-2.04)** | **2.40 (1.60-3.61)** | **2.71 (1.72-4.26)** |
| **CMM combined with social activity** |  |  |  |  |
| No CMD & socially active | 1.00 (reference) | 1.00 (reference) | 1.00 (reference) | 1.00 (reference) |
| No CMD & socially inactive | 1.00 (reference) | **1.62 (1.31-2.00)** | **1.94 (1.48-2.55)** | **2.40 (1.69-3.41)** |
| Single CMD & socially active | 1.00 (reference) | 1.08 (0.86-1.34) | **1.49 (1.10-2.01)** | **1.77 (1.22-2.57)** |
| Single CMD & socially inactive | 1.00 (reference) | **1.63 (1.24-2.13)** | **2.93 (2.12-4.06)** | **3.94 (2.64-5.87)** |
| CMM & socially active | 1.00 (reference) | 1.41 (0.90-2.20) | 1.61 (0.89-2.90) | **1.96 (1.01-3.81)** |
| CMM & socially inactive | 1.00 (reference) | **2.54 (1.42-4.56)** | **6.50 (3.50-12.1)** | **8.80 (4.47-17.3)** |

Covariates include cohort, sex, educational level, total household wealth, marital status, body mass index, current smoking status, alcohol consumption, physical activity, baseline hypertension, lung diseases, and cancer. For the model of baseline CMM, social activity was additionally adjusted.

Odds ratios and 95% confidence intervals were reported in the table. CMD: cardiometabolic disease; CMM: cardiometabolic multimorbidity.

## **Table S11.** Sensitivity analysis for the association of cardiometabolic multimorbidity and social activity with joint trajectories of physical disability, depressive symptom, and cognitive function by excluding participants with missing covariates.

|  | **Favourable trajectories of physical disability, depressive symptom and cognitive function** | **Favourable trajectories of physical disability and depressive symptom, and worsening cognitive function** | **Favourable trajectory of physical disability, and worsening depressive symptom and cognitive function** | **Rapidly-worsening physical disability and worsening depressive symptom and cognitive function** |
| --- | --- | --- | --- | --- |
| **CMM status at baseline** |  |  |  |  |
| No CMD | 1.00 (reference) | 1.00 (reference) | 1.00 (reference) | 1.00 (reference) |
| Single CMD | 1.00 (reference) | **1.11 (1.03-1.19)** | **1.65 (1.52-1.79)** | **2.08 (1.83-2.36)** |
| CMM | 1.00 (reference) | **1.30 (1.12-1.52)** | **2.40 (2.03-2.83)** | **3.39 (2.74-4.21)** |
| **CMM combined with social activity** | |  |  |  |
| No CMD & socially active | 1.00 (reference) | 1.00 (reference) | 1.00 (reference) | 1.00 (reference) |
| No CMD & socially inactive | 1.00 (reference) | **1.52 (1.43-1.61)** | **1.74 (1.60-1.89)** | **1.97 (1.69-2.29)** |
| Single CMD & socially active | 1.00 (reference) | **1.14 (1.04-1.25)** | **1.69 (1.50-1.90)** | **2.14 (1.77-2.58)** |
| Single CMD & socially inactive | 1.00 (reference) | **1.62 (1.47-1.79)** | **2.78 (2.47-3.13)** | **3.96 (3.30-4.75)** |
| CMM & socially active | 1.00 (reference) | **1.29 (1.05-1.59)** | **2.34 (1.84-2.97)** | **3.16 (2.29-4.36)** |
| CMM & socially inactive | 1.00 (reference) | **2.02 (1.62-2.51)** | **4.29 (3.40-5.42)** | **7.08 (5.28-9.49)** |

Covariates include cohort, age at baseline, sex, educational level, total household wealth, marital status, body mass index, current smoking status, alcohol consumption, physical activity, baseline hypertension, lung diseases, and cancer. For the model of baseline CMM, social activity was additionally adjusted.

Odds ratios and 95% confidence intervals were reported in the table. CMD: cardiometabolic disease; CMM: cardiometabolic multimorbidity.

## **Table S12.** Sensitivity analysis for the association of cardiometabolic multimorbidity and social activity with joint trajectories of physical disability, depressive symptom, cognitive function by imputing missing covariates.

|  | **Favourable trajectories of physical disability, depressive symptom and cognitive function** | **Favourable trajectories of physical disability and depressive symptom, and worsening cognitive function** | **Favourable trajectory of physical disability, and worsening depressive symptom and cognitive function** | | **Rapidly-worsening physical disability and worsening depressive symptom and cognitive function** | |
| --- | --- | --- | --- | --- | --- | --- |
| **CMM status at baseline** |  |  |  | |  | |
| No CMD | 1.00 (reference) | 1.00 (reference) | 1.00 (reference) | | 1.00 (reference) | |
| Single CMD | 1.00 (reference) | **1.09 (1.02-1.17)** | **1.67 (1.54-1.80)** | | **2.10 (1.87-2.36)** | |
| CMM | 1.00 (reference) | **1.33 (1.15-1.97)** | **2.54 (2.17-2.97)** | | **3.44 (2.81-4.22)** | |
| **CMM combined with social activity** | |  |  |  | |  |
| No CMD & socially active | 1.00 (reference) | 1.00 (reference) | 1.00 (reference) | | 1.00 (reference) | |
| No CMD & socially inactive | 1.00 (reference) | **1.51 (1.42-1.59)** | **1.75 (1.62-1.89)** | | **2.02 (1.77-2.32)** | |
| Single CMD & socially active | 1.00 (reference) | **1.11 (1.02-1.21)** | **1.72 (1.54-1.93)** | | **2.19 (1.85-2.60)** | |
| Single CMD & socially inactive | 1.00 (reference) | **1.61 (1.46-1.77)** | **2.82 (2.52-3.15)** | | **4.07 (3.44-4.81)** | |
| CMM & socially active | 1.00 (reference) | **1.28 (1.05-1.55)** | **2.45 (1.96-3.07)** | | **3.25 (2.41-4.37)** | |
| CMM & socially inactive | 1.00 (reference) | **2.12 (1.72-2.61)** | **4.65 (3.72-5.81)** | | **7.40 (5.61-9.78)** | |

Covariates include cohort, age at baseline, sex, educational level, total household wealth, marital status, body mass index, current smoking status, alcohol consumption, physical activity, baseline hypertension, lung diseases, and cancer. For the model of baseline CMM, social activity was additionally adjusted.

Odds ratios and 95% confidence intervals were reported in the table. CMD: cardiometabolic disease; CMM: cardiometabolic multimorbidity.

## **Table S13.** Sensitivity analysis for the association of cardiometabolic multimorbidity and social activity with joint trajectories of physical disability, depressive symptom, and cognitive function by including treatments or medications for hypertension, diabetes and heart problems in the models.

|  | **Favourable trajectories of physical disability, depressive symptom and cognitive function** | **Favourable trajectories of physical disability and depressive symptom, and worsening cognitive function** | **Favourable trajectory of physical disability, and worsening depressive symptom and cognitive function** | **Rapidly-worsening physical disability and worsening depressive symptom and cognitive function** |
| --- | --- | --- | --- | --- |
| **CMM status at baseline** |  |  |  |  |
| No CMD | 1.00 (reference) | 1.00 (reference) | 1.00 (reference) | 1.00 (reference) |
| Single CMD | 1.00 (reference) | 1.00 (0.92-1.09) | **1.48 (1.34-1.63)** | **1.94 (1.69-2.23)** |
| CMM | 1.00 (reference) | 1.12 (0.94-1.33) | **2.01 (1.65-2.44)** | **2.99 (2.32-3.86)** |
| **CMM combined with social activity** | |  |  |  |
| No CMD & socially active | 1.00 (reference) | 1.00 (reference) | 1.00 (reference) | 1.00 (reference) |
| No CMD & socially inactive | 1.00 (reference) | **1.51 (1.42-1.59)** | **1.74 (1.61-1.88)** | **1.99 (1.74-2.28)** |
| Single CMD & socially active | 1.00 (reference) | 1.02 (0.92-1.13) | **1.53 (1.35-1.73)** | **2.03 (1.69-2.45)** |
| Single CMD & socially inactive | 1.00 (reference) | **1.47 (1.32-1.63)** | **2.48 (2.19-2.81)** | **3.69 (3.07-4.43)** |
| CMM & socially active | 1.00 (reference) | 1.07 (0.85-1.33) | **1.94 (1.50-2.50)** | **2.82 (2.01-3.94)** |
| CMM & socially inactive | 1.00 (reference) | **1.78 (1.41-2.24)** | **3.65 (2.84-4.70)** | **6.31 (4.59-8.68)** |

Covariates include cohort, age at baseline, sex, educational level, total household wealth, marital status, body mass index, current smoking status, alcohol consumption, physical activity, baseline hypertension, lung diseases, and cancer, and medications for hypertension, diabetes and heart problems. For the model of baseline CMM, social activity was additionally adjusted.

Odds ratios and 95% confidence intervals were reported in the table. CMD: cardiometabolic disease; CMM: cardiometabolic multimorbidity.

## **Table S14.** Sensitivity analysis for the association of cardiometabolic multimorbidity and social activity with joint trajectories of physical disability, depressive symptom, and cognitive function by redefining social activity as respondents participated at least one social activities or groups in last month or year.

|  | **Favourable trajectories of physical disability, depressive symptom and cognitive function** | **Favourable trajectories of physical disability and depressive symptom, and worsening cognitive function** | **Favourable trajectory of physical disability, and worsening depressive symptom and cognitive function** | **Rapidly-worsening physical disability and worsening depressive symptom and cognitive function** |
| --- | --- | --- | --- | --- |
| **CMM status at baseline** |  |  |  |  |
| No CMD | 1.00 (reference) | 1.00 (reference) | 1.00 (reference) | 1.00 (reference) |
| Single CMD | 1.00 (reference) | 1.00 (0.92-1.09) | **1.48 (1.34-1.63)** | **1.94 (1.69-2.23)** |
| CMM | 1.00 (reference) | 1.12 (0.94-1.33) | **2.01 (1.65-2.44)** | **2.99 (2.32-3.86)** |
| **CMM combined with social activity** | |  |  |  |
| No CMD & socially active | 1.00 (reference) | 1.00 (reference) | 1.00 (reference) | 1.00 (reference) |
| No CMD & socially inactive | 1.00 (reference) | **1.10 (1.04-1.16)** | **1.20 (1.11-1.29)** | **1.40 (1.23-1.60)** |
| Single CMD & socially active | 1.00 (reference) | 1.02 (0.93-1.13) | **1.50 (1.34-1.69)** | **1.95 (1.64-2.32)** |
| Single CMD & socially inactive | 1.00 (reference) | 1.06 (0.95-1.19) | **1.73 (1.52-1.96)** | **2.71 (2.26-3.25)** |
| CMM & socially active | 1.00 (reference) | 1.12 (0.91-1.38) | **2.15 (1.71-2.71)** | **3.33 (2.46-4.51)** |
| CMM & socially inactive | 1.00 (reference) | 1.23 (0.96-1.57) | **2.20 (1.68-2.89)** | **3.75 (2.66-5.28)** |

Covariates include cohort, age at baseline, sex, educational level, total household wealth, marital status, body mass index, current smoking status, alcohol consumption, physical activity, baseline hypertension, lung diseases, and cancer, and medications for hypertension, diabetes and heart problems. For the model of baseline CMM, social activity was additionally adjusted.

Odds ratios and 95% confidence intervals were reported in the table. CMD: cardiometabolic disease; CMM: cardiometabolic multimorbidity.

## **Table S15.** Baseline characteristics of participants according to whether died during follow-up.

|  | **Total**  **(N=79997)** | **Whether died during follow-up** | | **P value** |
| --- | --- | --- | --- | --- |
|  |  | **No**  **(N=70410)** | **Yes**  **(N=9587)** |  |
| **Study** |  |  |  | <0.001 |
| CHARLS | 13902 (17.4) | 13667 (19.4) | 235 (2.5) |  |
| ELSA | 8236 (10.3) | 8205 (11.7) | 31 (0.3) |  |
| HRS | 4484 (5.6) | 0 (0.0) | 4484 (46.8) |  |
| SHARE | 53375 (66.7) | 48538 (68.9) | 4837 (50.5) |  |
| **Age at baseline, mean (SD)** | 63.7 (9.2) | 63.2 (9.1) | 68.0 (8.8) | <0.001 |
| **Sex, n (%)** |  |  |  | 0.002 |
| Male | 35290 (44.1) | 31204 (44.3) | 4086 (42.6) |  |
| Female | 44707 (55.9) | 39206 (55.7) | 5501 (57.4) |  |
| **Educational level, n (%)** |  |  |  | <0.001 |
| Primary | 21125 (26.4) | 19447 (27.6) | 1678 (17.5) |  |
| Secondary | 40891 (51.1) | 36198 (51.4) | 4693 (49.0) |  |
| Tertiary | 17338 (21.7) | 14125 (20.1) | 3213 (33.5) |  |
| Unknown | 643 (0.8) | 640 (0.9) | 3 (0.0) |  |
| **Total household wealth, n (%)** |  |  |  | <0.001 |
| Q1 (lowest) | 18663 (23.3) | 16606 (23.6) | 2057 (21.5) |  |
| Q2 | 19518 (24.4) | 16618 (23.6) | 2900 (30.2) |  |
| Q3 | 19029 (23.8) | 16608 (23.6) | 2421 (25.3) |  |
| Q4 (highest) | 18755 (23.4) | 16609 (23.6) | 2146 (22.4) |  |
| Unknown | 4032 (5.0) | 3969 (5.6) | 63 (0.7) |  |
| **Marital status, n (%)** |  |  |  | <0.001 |
| Married or partnered | 62959 (78.7) | 56119 (79.7) | 6840 (71.3) |  |
| Widowed | 8389 (10.5) | 6933 (9.8) | 1456 (15.2) |  |
| Separated or divorced or single | 8591 (10.7) | 7307 (10.4) | 1284 (13.4) |  |
| Unknown | 58 (0.1) | 51 (0.1) | 7 (0.1) |  |
| **Body mass index, kg/m^2^, n (%)** |  |  |  | <0.001 |
| <18.5 | 1347 (1.7) | 1230 (1.7) | 117 (1.2) |  |
| 18.5-24.9 | 29410 (36.8) | 26464 (37.6) | 2946 (30.7) |  |
| 25-29.9 | 29624 (37.0) | 25868 (36.7) | 3756 (39.2) |  |
| ≥30 | 15395 (19.2) | 12820 (18.2) | 2575 (26.9) |  |
| Unknown | 4221 (5.3) | 4028 (5.7) | 193 (2.0) |  |
| **Current smoking status, n (%)** |  |  |  | <0.001 |
| No | 64633 (80.8) | 56400 (80.1) | 8233 (85.9) |  |
| Yes | 15326 (19.2) | 13999 (19.9) | 1327 (13.8) |  |
| Unknown | 38 (0.0) | 11 (0.0) | 27 (0.3) |  |
| **Alcohol consumption, n (%)** |  |  |  | <0.001 |
| Less than weekly drinking | 43834 (54.8) | 38134 (54.2) | 5700 (59.5) |  |
| Weekly drinking or more | 35204 (44.0) | 31345 (44.5) | 3859 (40.3) |  |
| Unknown | 959 (1.2) | 931 (1.3) | 28 (0.3) |  |
| **Physical activity, n (%)** |  |  |  | <0.001 |
| Physical inactive | 15767 (19.7) | 14292 (20.3) | 1475 (15.4) |  |
| Physical active | 64226 (80.3) | 56115 (79.7) | 8111 (84.6) |  |
| Unknown | 4 (0.0) | 3 (0.0) | 1 (0.0) |  |
| **Baseline comorbidities, n (%)** |  |  |  |  |
| Hypertension | 32738 (40.9) | 27473 (39.0) | 5265 (54.9) | <0.001 |
| Cancer | 5945 (7.4) | 4672 (6.6) | 1273 (13.3) | <0.001 |
| Lung disease | 6198 (7.7) | 5289 (7.5) | 909 (9.5) | <0.001 |
| **Cardiometabolic multimorbidity, n (%)** |  |  |  | <0.001 |
| No cardiometabolic disease | 59929 (74.9) | 53902 (76.6) | 6027 (62.9) |  |
| Single cardiometabolic disease | 16349 (20.4) | 13677 (19.4) | 2672 (27.9) |  |
| Cardiometabolic multimorbidity | 3719 (4.6) | 2831 (4.0) | 888 (9.3) |  |

# Supplementary Figures


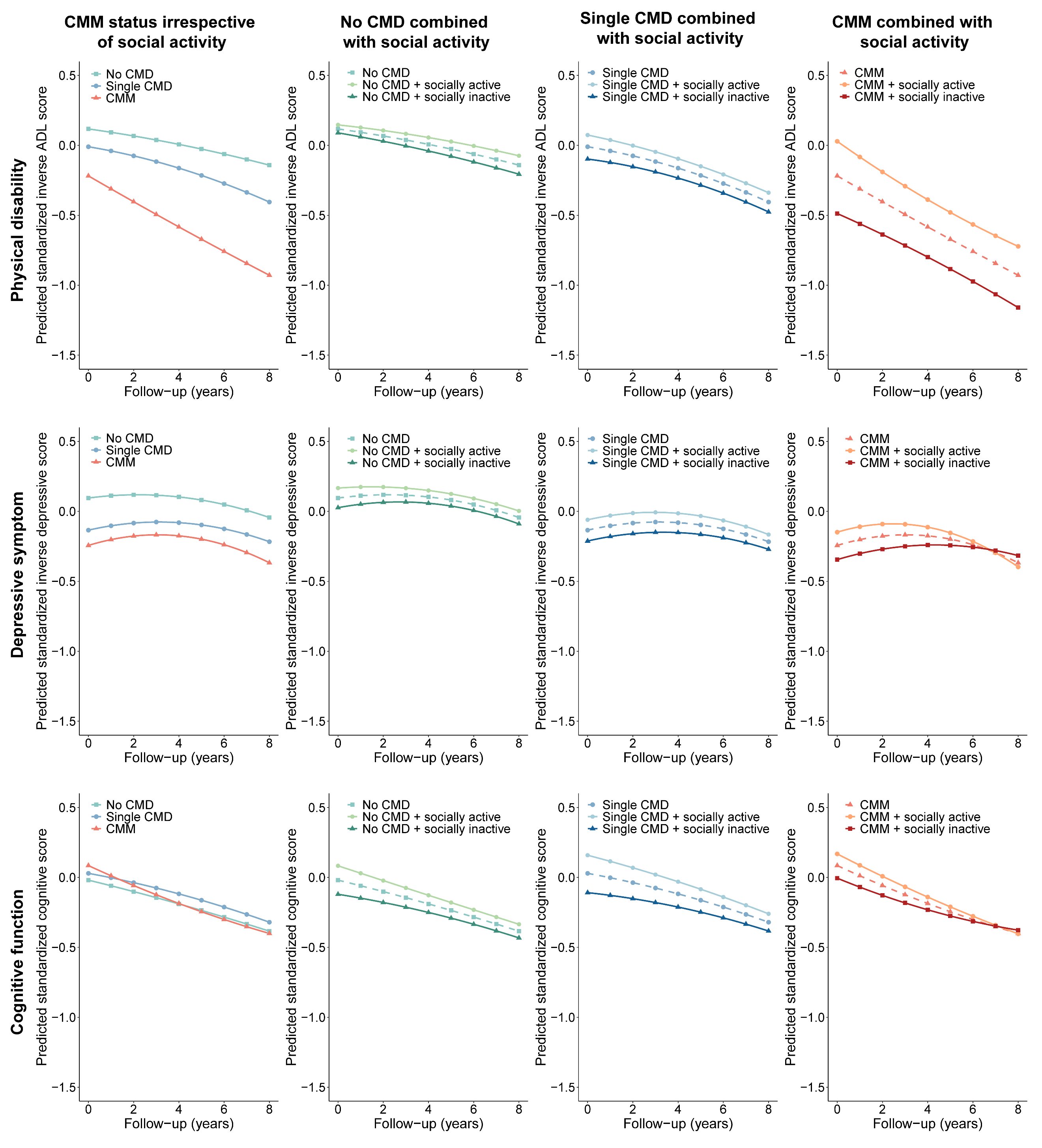


## **Figure S1.** Estimated trajectories of physical disability, depressive symptom, and cognitive function during follow-up by cardiometabolic multimorbidity and social activity in CHARLS.

Trajectories are adjusted for age at baseline, sex, educational level, total household wealth, marital status, body mass index, current smoking status, alcohol consumption, physical activity, baseline hypertension, lung diseases, and cancer.

Lower values of inverse ADL score, inverse depressive score, and cognitive score indicated worse physical disability, depressive symptoms, and cognitive function. ADL, activities of daily living; CMD, cardiometabolic disease; CMM, cardiometabolic multimorbidity.


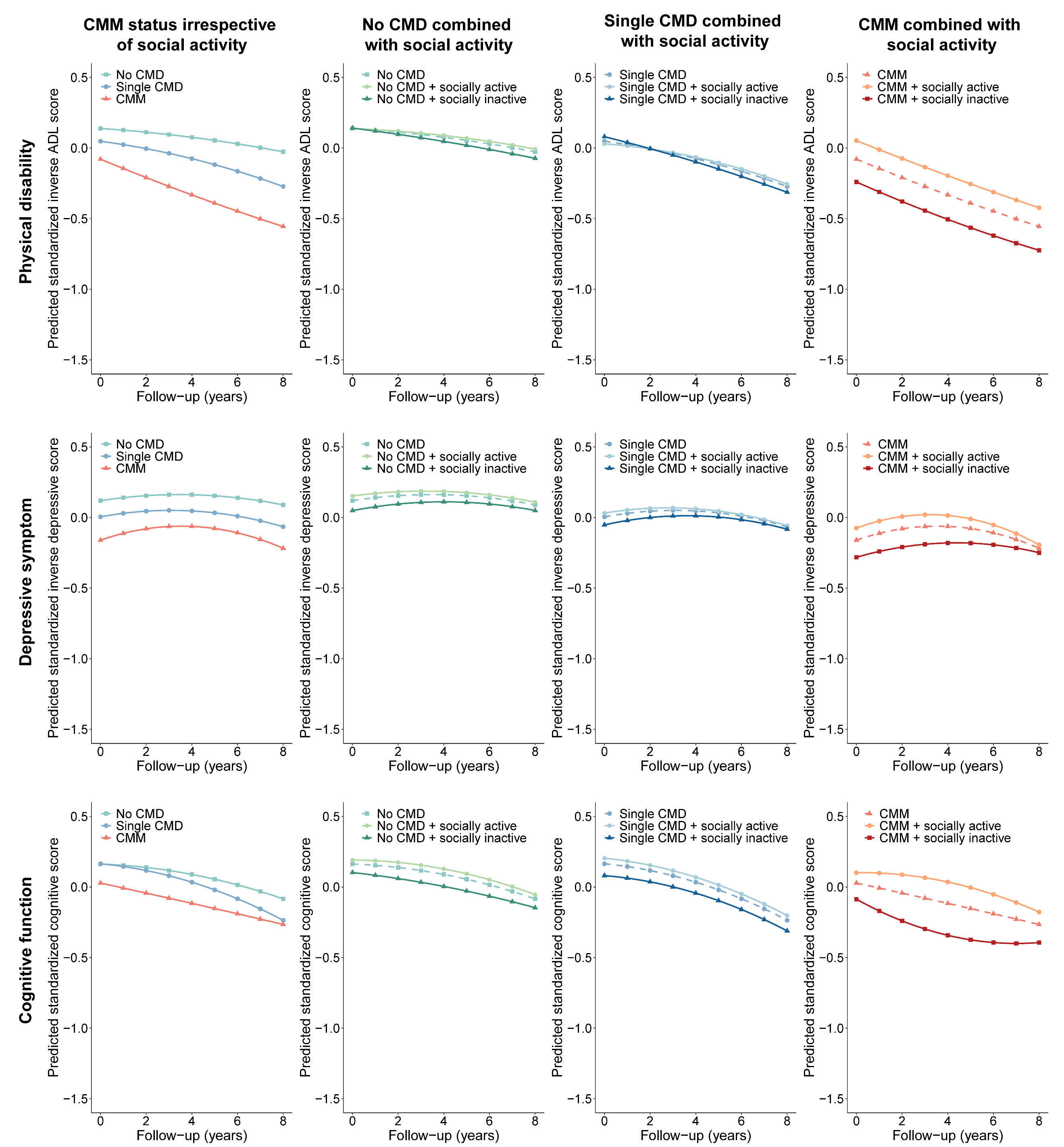


## **Figure S2.** Estimated trajectories of physical disability, depressive symptom, and cognitive function during follow-up by cardiometabolic multimorbidity and social activity in ELSA.

Trajectories are adjusted for age at baseline, sex, educational level, total household wealth, marital status, body mass index, current smoking status, alcohol consumption, physical activity, baseline hypertension, lung diseases, and cancer.

Lower values of inverse ADL score, inverse depressive score, and cognitive score indicated worse physical disability, depressive symptoms, and cognitive function. ADL, activities of daily living; CMD, cardiometabolic disease; CMM, cardiometabolic multimorbidity.


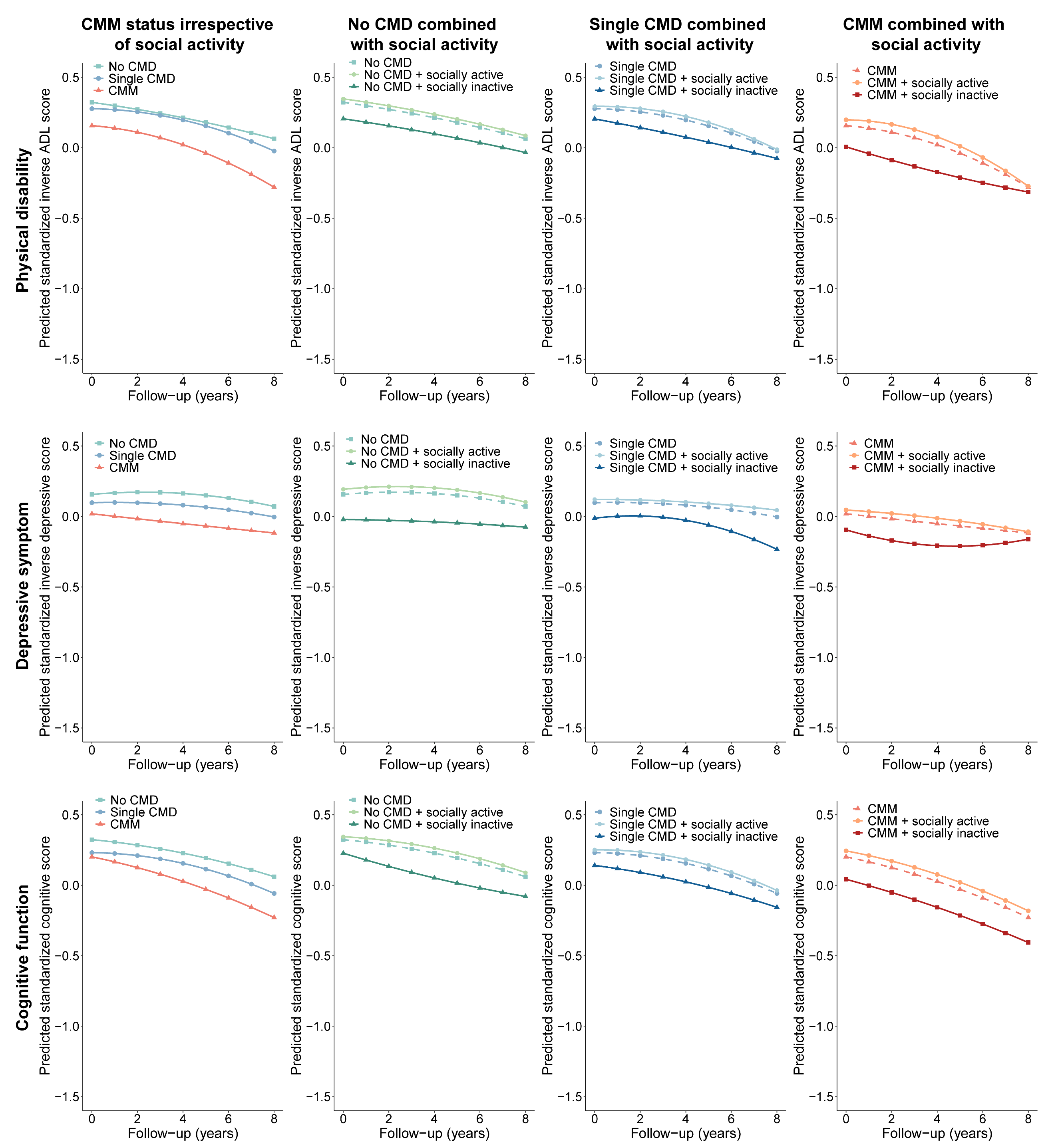


## **Figure S3.** Estimated trajectories of physical disability, depressive symptom, and cognitive function during follow-up by cardiometabolic multimorbidity and social activity in HRS.

Trajectories are adjusted for age at baseline, sex, educational level, total household wealth, marital status, body mass index, current smoking status, alcohol consumption, physical activity, baseline hypertension, lung diseases, and cancer.

Lower values of inverse ADL score, inverse depressive score, and cognitive score indicated worse physical disability, depressive symptoms, and cognitive function. ADL, activities of daily living; CMD, cardiometabolic disease; CMM, cardiometabolic multimorbidity.

**
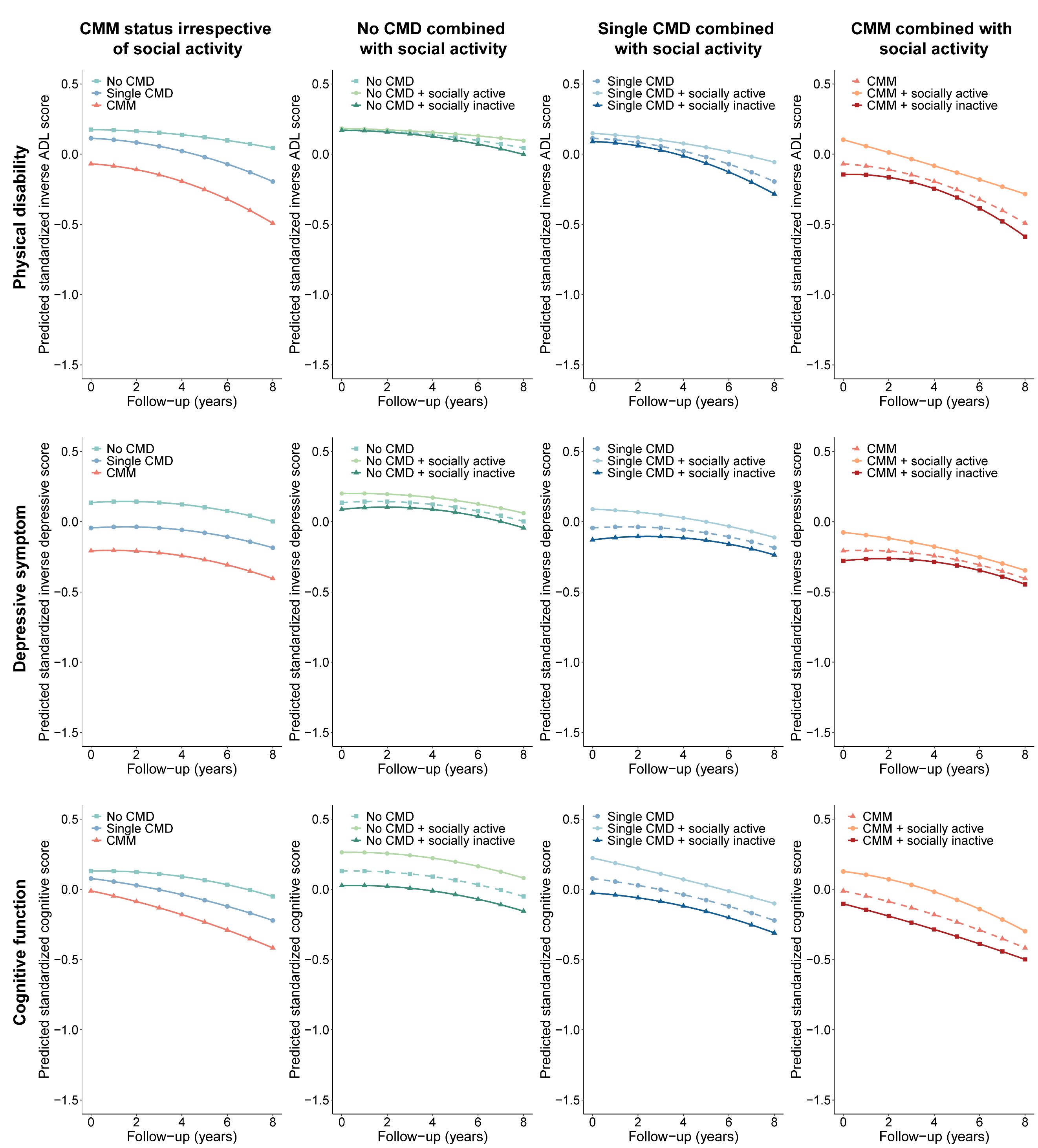
**

## **Figure S4.** Estimated trajectories of physical disability, depressive symptom, and cognitive function during follow-up by cardiometabolic multimorbidity and social activity in SHARE.

Trajectories are adjusted for age at baseline, sex, educational level, total household wealth, marital status, body mass index, current smoking status, alcohol consumption, physical activity, baseline hypertension, lung diseases, and cancer.

Lower values of inverse ADL score, inverse depressive score, and cognitive score indicated worse physical disability, depressive symptoms, and cognitive function. ADL, activities of daily living; CMD, cardiometabolic disease; CMM, cardiometabolic multimorbidity.


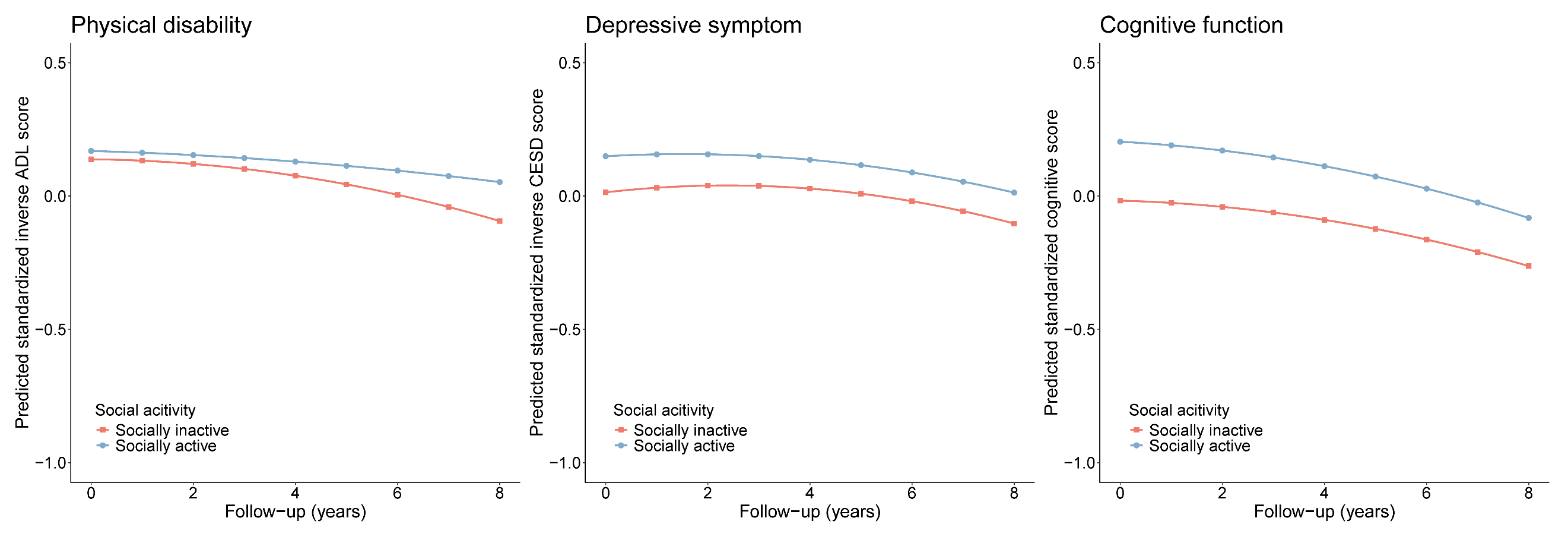


## **Figure S5.** Predicted values of physical disability, depressive symptom, and cognitive function during follow-up stratified by social activity among the combined population.

Covariates include cohort, age at baseline, sex, educational level, total household wealth, marital status, body mass index, current smoking status, alcohol consumption, physical activity, baseline hypertension, lung diseases, and cancer.


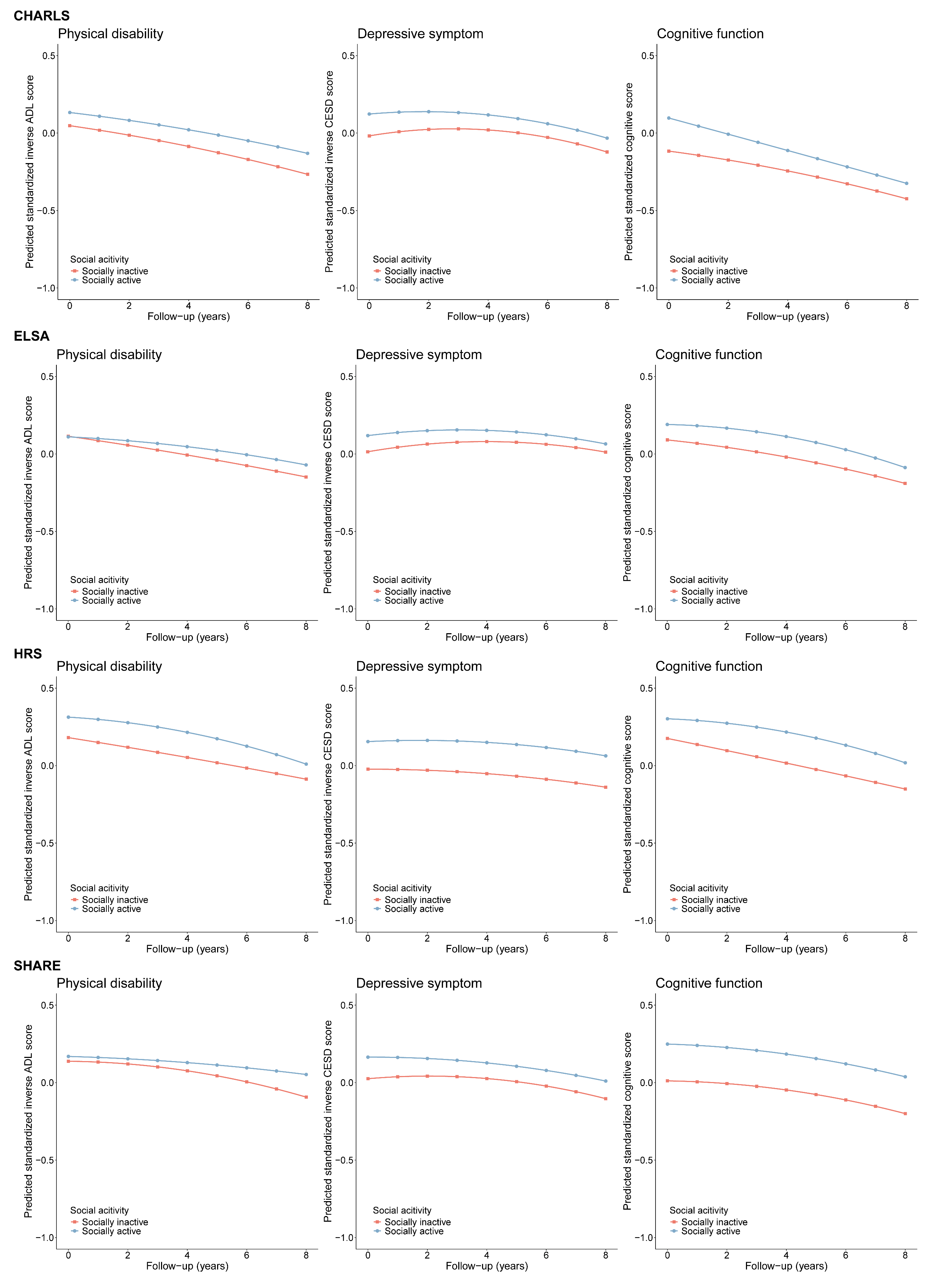


## **Figure S6.** Estimated trajectories of physical disability, depressive symptom, and cognitive function during follow-up by cardiometabolic multimorbidity and social activity across different cohorts.

Covariates include age at baseline, sex, educational level, total household wealth, marital status, body mass index, current smoking status, alcohol consumption, physical activity, baseline hypertension, lung diseases, and cancer.


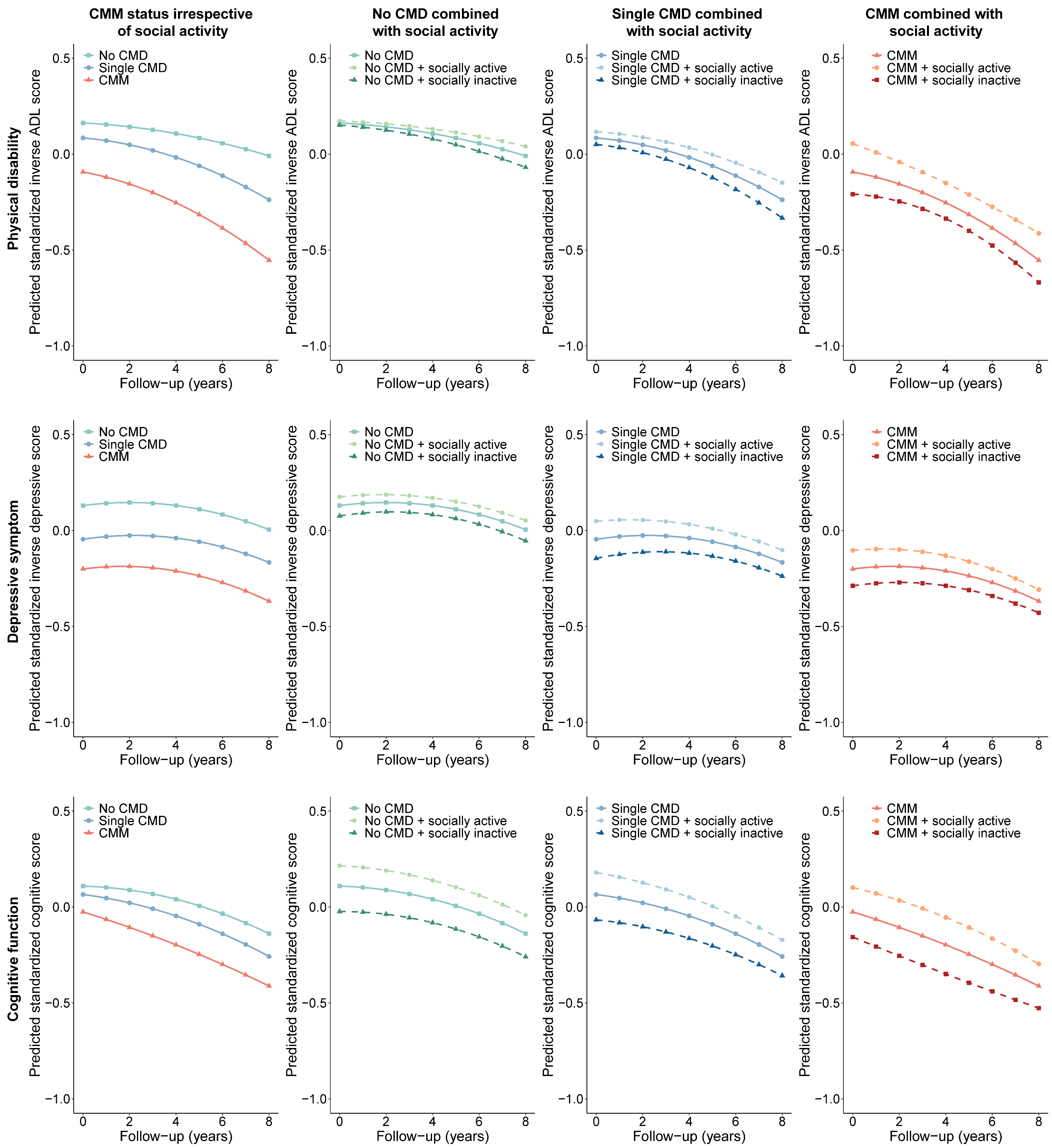


## **Figure S7**. Sensitivity analysis for estimated trajectories of physical disability, depressive symptoms, and cognitive function during follow-up by cardiometabolic multimorbidity and social activity by redefining social activity as respondents participated at least one social activities or groups in last month or year.

Trajectories are adjusted for age at baseline, sex, educational level, total household wealth, marital status, body mass index, current smoking status, alcohol consumption, physical activity, baseline hypertension, lung diseases, and cancer.

Lower values of inverse ADL score, inverse depressive score, and cognitive score indicated worse physical disability, depressive symptom, and cognitive function. ADL, activities of daily living; CMD, cardiometabolic disease; CMM, cardiometabolic multimorbidity.


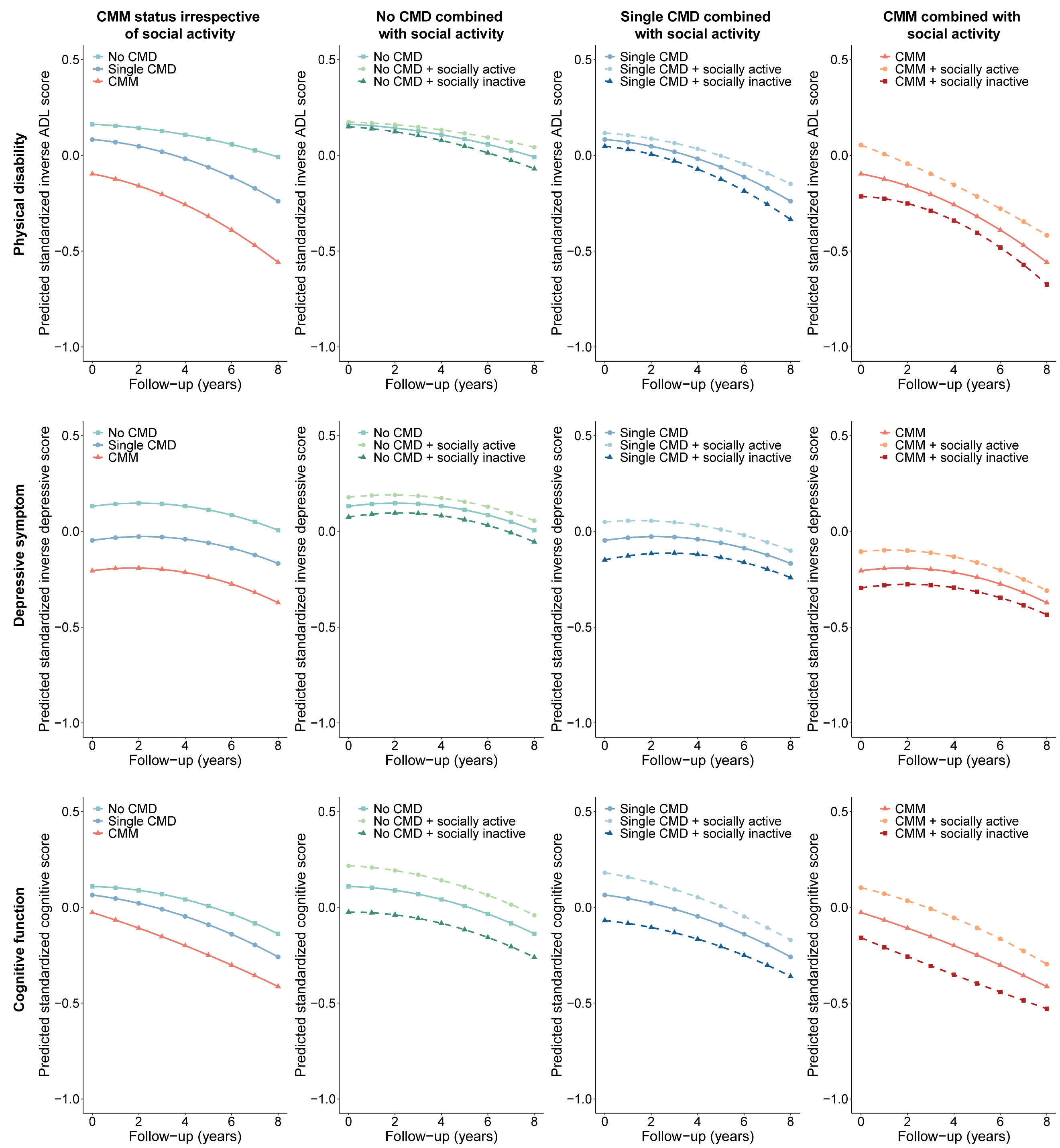


## **Figure S8.** Sensitivity analysis for estimated trajectories of physical disability, depressive symptoms, and cognitive function during follow-up by cardiometabolic multimorbidity and social activity by imputing missing covariates.

Trajectories are adjusted for age at baseline, sex, educational level, total household wealth, marital status, body mass index, current smoking status, alcohol consumption, physical activity, baseline hypertension, lung diseases, and cancer.

Lower values of inverse ADL score, inverse depressive score, and cognitive score indicated worse physical disability, depressive symptoms and cognitive function. ADL, activities of daily living; CMD, cardiometabolic disease; CMM, cardiometabolic multimorbidity.


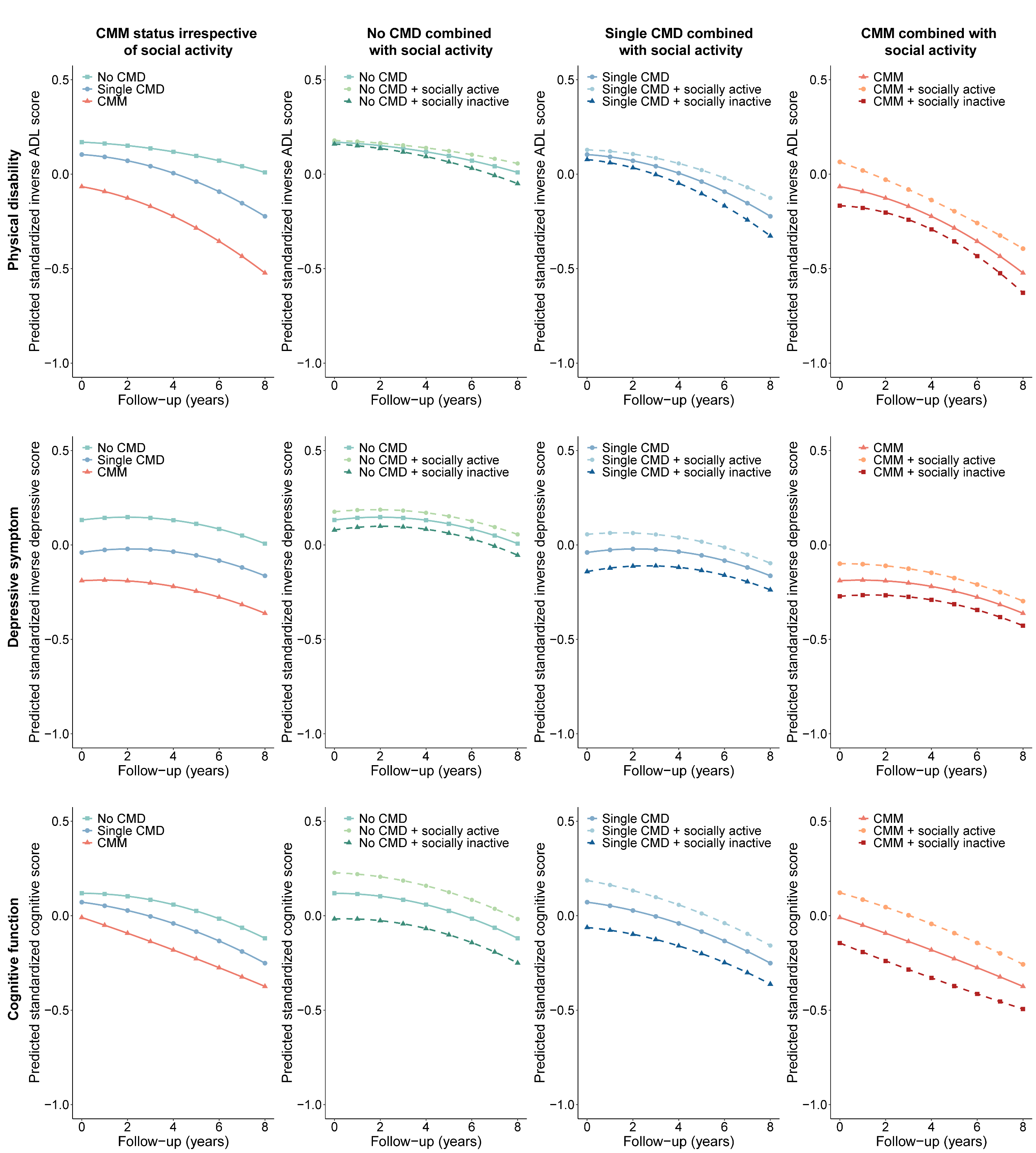


## **Figure S9.** Sensitivity analysis for estimated trajectories of physical disability, depressive symptoms, and cognitive function during follow-up by cardiometabolic multimorbidity and social activity by deleting participants with missing covariates.

Trajectories are adjusted for age at baseline, sex, educational level, total household wealth, marital status, body mass index, current smoking status, alcohol consumption, physical activity, baseline hypertension, lung diseases, and cancer.

Lower values of inverse ADL score, inverse depressive score, and cognitive score indicated worse physical disability, depressive symptoms and cognitive function. ADL, activities of daily living; CMD, cardiometabolic disease; CMM, cardiometabolic multimorbidity.


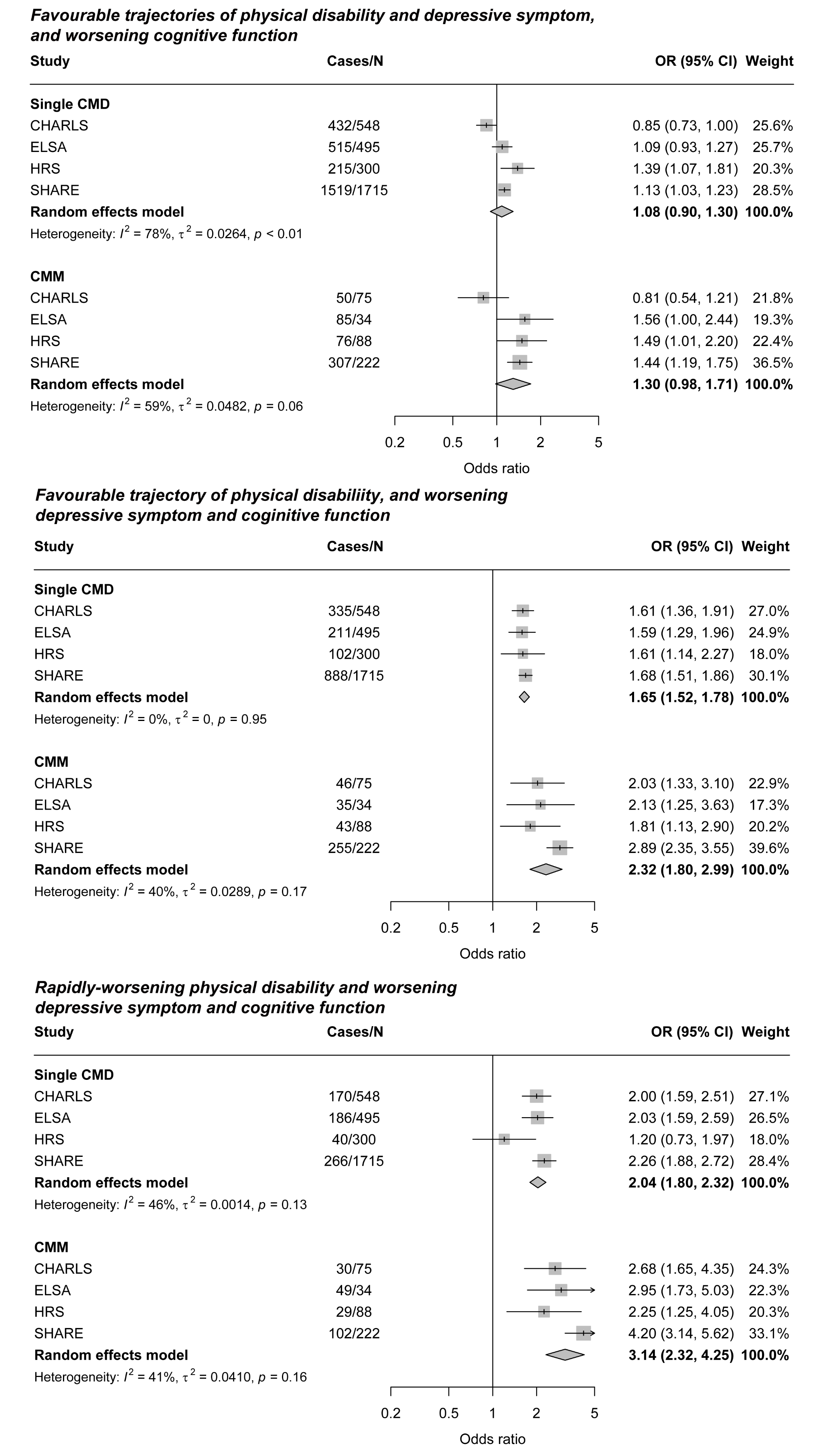


## **Figure S10.** Random effect meta-analyses for the association of CMM with the joint trajectories of physical disability, depressive symptom and cognitive function.

The trajectory of favourable and stable physical disability, depressive symptom, and cognitive function, and no CMD is the reference.

Covariates include age at baseline, sex, educational level, total household wealth, marital status, body mass index, current smoking status, alcohol consumption, physical activity, social activity, and baseline hypertension and cancer.
